# Protonation state of the Cu_4_S_2_ Cu_Z_ site in nitrous oxide reductase: redox dependence and insight into reactivity†
†Electronic supplementary information (ESI) available: Full experimental and computational methodology, EPR quantification of the Cu*Z/Cu_Z_ ratio, 2nd derivative of the X band EPR of 1-hole Cu_Z_, table of absorption band energies and assignments for 1-hole Cu_Z_ and 1-hole Cu*Z, resonance Raman spectrum and profile of 1-hole Cu*Z, pH dependence of the spectral features of 1-hole and 2-hole Cu_Z_, low temperature absorption spectrum of 2-hole Cu_Z_, and computational structures, vibrations, and TD-DFT absorption spectra for models of 1-hole and 2-hole Cu_Z_ (1-hole SH^–^ and OH^–^ and 2-hole SH^–^ and S^2–^ models). See DOI: 10.1039/c5sc02102b


**DOI:** 10.1039/c5sc02102b

**Published:** 2015-07-03

**Authors:** Esther M. Johnston, Simone Dell'Acqua, Sofia R. Pauleta, Isabel Moura, Edward I. Solomon

**Affiliations:** a Department of Chemistry , Stanford University , Stanford , CA 94305 , USA . Email: edward.solomon@stanford.edu; b Dipartimento di Chimica , Università di Pavia , Via Taramelli 12 , 27100 Pavia , Italy; c UCIBIO , REQUIMTE , Departamento de Química , Faculdade de Ciências e Tecnologia , Universidade Nova de Lisboa , 2829-516 Caparica , Portugal

## Abstract

Spectroscopic and computational methods have been used to determine the protonation state of the edge sulfur ligand in the Cu_4_S_2_ Cu_Z_ form of the active site of nitrous oxide reductase (N_2_OR) in its 3Cu^I^Cu^II^ (1-hole) and 2Cu^I^2Cu^II^ (2-hole) redox states. The EPR, absorption, and MCD spectra of 1-hole Cu_Z_ indicate that the unpaired spin in this site is evenly delocalized over Cu_I_, Cu_II_, and Cu_IV_. 1-hole Cu_Z_ is shown to have a μ_2_-thiolate edge ligand from the observation of S–H bending modes in the resonance Raman spectrum at 450 and 492 cm^–1^ that have significant deuterium isotope shifts (–137 cm^–1^) and are not perturbed up to pH 10. 2-hole Cu_Z_ is characterized with absorption and resonance Raman spectroscopies as having two Cu–S stretching vibrations that profile differently. DFT models of the 1-hole and 2-hole Cu_Z_ sites are correlated to these spectroscopic features to determine that 2-hole Cu_Z_ has a μ_2_-sulfide edge ligand at neutral pH. The slow two electron (+1 proton) reduction of N_2_O by 1-hole Cu_Z_ is discussed and the possibility of a reaction between 2-hole Cu_Z_ and O_2_ is considered.

## Abstract 2

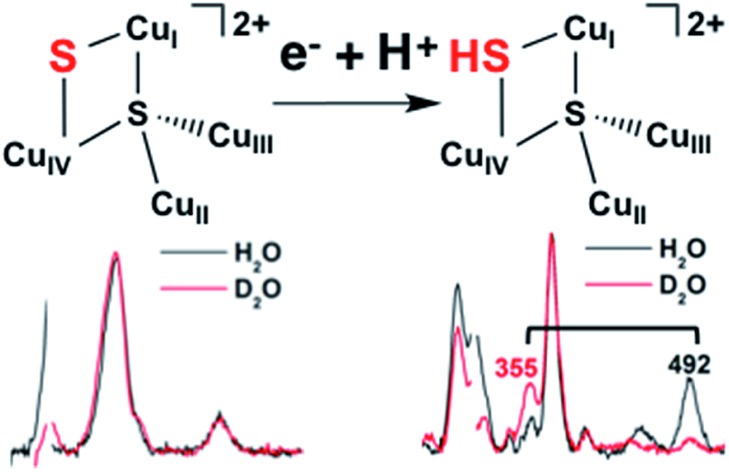
The edge ligand in the Cu_4_S_2_ Cu_Z_ form of nitrous oxide reductase is a μ_2_-thiolate in the 1-hole and a μ_2_-sulfide in the 2-hole redox state, leading to proton-coupled electron transfer reactivity.

## 1. Introduction

The main reductive part of the nitrogen cycle, known as bacterial denitrification, is performed by soil and marine bacteria as a means of anaerobic or microaerobic respiration. Denitrification involves the conversion of nitrate to dinitrogen *via* four successive reductive steps (NO_3_^–^ → NO_2_^–^ → NO → N_2_O → N_2_), each performed by a different metalloenzyme.^1^ The terminal product of denitrification can be either N_2_O or N_2_, depending on the regulatory control of the N_2_O reduction process and whether the bacterium involved contains the gene cluster for nitrous oxide reduction (the *nos* cluster; *nosZ* encodes the nitrous oxide reductase enzyme).^2^,^3^ The N_2_O reduction process and its regulation *in vivo* are of significant interest because N_2_O is a potent greenhouse gas, with a global warming potential 300× that of CO_2_,^4^,^5^ and depletes the ozone layer.^6^ Anthropogenic sources of environmental N_2_O, the majority of which is due to agricultural activity, is an increasing contribution to the global atmosphere.^2^ Soil studies have indicated that pH,^7^,^8^ temperature,^9^ acetylene,^10^ sulfide,^11^ and dioxygen^12^ all affect the production of N_2_O, but the molecular basis of these effects is still not known. A molecular understanding of nitrous oxide reduction and how this process is regulated could enable mitigation of N_2_O release from anthropogenic sources.^5^

**Fig. 1:**
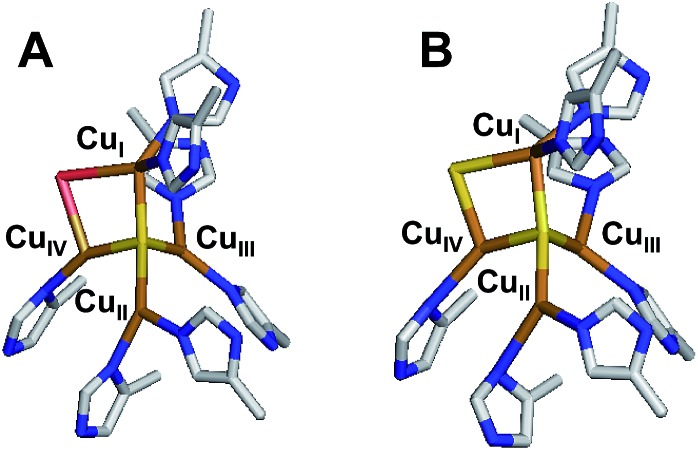
The two forms of the tetranuclear copper sulfide active site of nitrous oxide reductase. (A) Cu*Z in *Pd*N_2_OR isolated aerobically (PDB ID ; 1FWX).^13^ (B) Cu_Z_ in *Ps*N_2_OR isolated anaerobically (PDB ID ; 3SBP).^14^

Nitrous oxide reductase contains two copper sites: a binuclear site known as Cu_A_ that functions as an electron transfer site, and an unusual tetranuclear copper sulfide cluster active site, where N_2_O binds and is reduced (Fig. 1). Two forms of this tetranuclear site have been structurally characterized. One, known as Cu*Z, has a μ_4_ sulfide ligand bridging all four coppers and a solvent derived ligand on an open edge (the Cu_I_–Cu_IV_ edge) where N_2_O is proposed to bind (Fig. 1A).^13^ This edge ligand has previously been assigned as a bridging hydroxide ligand, due to the presence of a vibration in the resonance Raman spectrum of Cu*Z that shifts in H_2_^18^O solvent at high pH and the absence of significant spectroscopic differences between Cu*Z at high and low pH.^15^ The other form of the cluster, known as Cu_Z_, has an additional μ_2_ sulfur ligand bridging the Cu_I_–Cu_IV_ edge (Fig. 1B).^14^ Whether the μ_2_ edge ligand in Cu_Z_ is a thiolate (SH^–^) or a sulfide (S^2–^) and how its protonation depends on the redox state of the cluster are not known. The Cu_4_S_2_ Cu_Z_ form of the cluster is dominantly isolated when N_2_OR is purified in the absence of oxygen^16^ or rapidly in the presence of oxygen,^17^ while the Cu_4_S Cu*Z form is isolated when the purification is performed aerobically or anaerobically from mutants in the accessory genes;^16^–^18^ however, all purifications typically yield enzyme with a mixture of the two sites.^17^,^19^ Which structural form of the cluster is responsible for N_2_O reduction *in vivo* is a matter of some debate.^20^–^22^ As isolated, neither N_2_OR containing a high percentage of Cu_Z_ nor N_2_OR containing a high percentage of Cu*Z shows high enough specific activity in steady-state assays to be consistent with N_2_OR activity in whole cells.^17^,^23^ N_2_OR containing Cu_Z_ can be activated by prolonged dialysis against base,^23^ while N_2_OR that contains Cu*Z can be reductively activated by preincubation with methyl viologen, which reduces Cu*Z to the active fully reduced (4Cu^I^) redox state.^24^,^25^ After activation, both Cu_Z_ and Cu*Z show specific activities consistent with whole cell N_2_OR activity.^20^,^23^ However, it has recently been shown that the Cu*Z site in its fully reduced redox state is the form of the cluster that is responsible for the rapid N_2_O reduction in steady state assays with methyl viologen, based on its rapid single turnover reaction with N_2_O. Alternatively, in single turnover studies Cu_Z_ in its 1-hole redox state reduces N_2_O but at a rate too slow to be catalytically relevant (10^–6^ that of the fully reduced state of Cu*Z).^26^ Thus, the physiological role of the Cu_Z_ site in nitrous oxide reduction and whether it participates in N_2_O reduction *in vivo* is unknown.

The Cu_Z_ site in nitrous oxide reductase has been extensively studied in N_2_OR isolated from *Pseudomonas stutzeri* (*Ps*N_2_OR)^27^,^28^ and *Paracoccus pantotrophus* (*Pp*N_2_OR).^19^,^29^ In the latter enzyme, Cu_Z_ has been shown to access two redox states, the resting 2Cu^I^2Cu^II^ (2-hole) redox state, and a 1 electron reduced 3Cu^I^Cu^II^ (1-hole) redox state (*E*^o^ = +60 mV).^19^ Both redox states of Cu_Z_ have previously been studied using EPR, absorption, MCD, and resonance Raman spectroscopies.^19^,^28^,^30^–^33^ However, these studies were performed before the elucidation of the presence of a second sulfur in the Cu_Z_ cluster, and so yielded limited direct insight into the cluster and the protonation state of the edge sulfur. Additionally, the previous studies were performed in the presence of background spectroscopic features from ∼30% Cu*Z, which complicates the analysis.^19^,^29^ These limitations lead to the conclusion that Cu_Z_ and Cu*Z were very similar and perhaps differed only in the second sphere.^28^ These results are now extended and correlated to the structural insight that Cu_Z_ contains an additional inorganic sulfur edge ligand.^14^ An understanding of the protonation state, electronic structure, and potential reactivity of the Cu_Z_ site is necessary to gain insight into its reactivity and role *in vivo*.

This study uses EPR, absorption, MCD and resonance Raman spectroscopies coupled with DFT calculations to determine the protonation state of the edge sulfur ligand in the 1-hole and 2-hole redox states of Cu_Z_ in *Marinobacter hydrocarbonoclasticus* N_2_OR (*Mh*N_2_OR) and to define the electronic structures of these states. This leads to insight into the nature of the reactivity of the 1-hole and 2-hole states of Cu_Z_ and the origin of the spectroscopic similarity between 1-hole Cu_Z_ and 1-hole Cu*Z, despite significant differences in edge ligation in the two sites.

## 2. Methodology

### 2.1 Summary of experimental methodology

Full experimental methodology and computational details can be found in the ESI,† while a summary is presented here. Nitrous oxide reductase (N_2_OR) was isolated from *Marinobacter hydrocarbonoclasticus* 617 (formerly *Pseudomonas nautica*) grown under microaerobic conditions in the presence of nitrate after two aerobic chromatographic steps without added reductant, as described previously.^17^ These purification conditions were shown to maximize the amount of Cu_4_S_2_ Cu_Z_ content relative to Cu_4_S Cu*Z in the purified enzyme. Samples containing larger amounts of Cu*Z were purified in parallel with three chromatographic purification steps from a batch of cells grown under anaerobic conditions in the presence of nitrate, and that had been stored at –80 °C for a long period.^17^,^26^ Both *Mh*N_2_OR samples showed copper quantitation results consistent with full occupancy of the Cu_A_ and Cu_Z_/Cu*Z sites (6.4 ± 0.2 and 6.2 ± 0.7 respectively). The percentage of Cu_Z_*versus* Cu*Z in the samples used for this study was determined by EPR spin quantitation (Fig. S1†). Samples purified with high amounts of Cu_Z_ contained 60 ± 10% Cu_Z_, while samples purified to obtain more Cu*Z contained 10 ± 10% Cu_Z_. Spectroscopic samples of 1-hole and 2-hole Cu_Z_ were prepared in a glove box under N_2_ atmosphere. Samples of 1-hole Cu_Z_ were prepared from *Mh*N_2_OR (60% Cu_Z_ and 40% Cu*Z) that had been incubated with 100 equivalents of reduced methyl viologen, with subsequent removal of the methyl viologen using a desalting column. Samples of 2-hole Cu_Z_ were prepared by reducing *Mh*N_2_OR (60 ± 10% Cu_Z_, 40 ± 10% Cu*Z) with 10 equivalents of sodium ascorbate, which reduces the Cu_A_ site rapidly and the 2-hole Cu_Z_ site very slowly, and spectra were collected within 1 hour so that minimal reduction of 2-hole Cu_Z_ was observed. In parallel, *Mh*N_2_OR samples containing 90 ± 10% Cu*Z were reduced with 10 equivalents of sodium ascorbate to obtain the spectral features of 1-hole Cu*Z. For pH and deuteration studies, samples of 1-hole and 2-hole Cu_Z_ were buffer exchanged by centrifugation into different pH or pD buffers. Typical *Mh*N_2_OR concentrations used for spectroscopic samples were 0.1–0.3 mM for absorption, MCD and EPR, and up to 0.5 mM for resonance Raman.

### 2.2 Computational modeling

A computational model of Cu_Z_ was built from the atomic coordinates of the crystal structure of *Pseudomonas stutzeri* N_2_OR, the only known structure of the Cu_4_S_2_ cluster (PDB ID ; 3SBP, resolution 1.7 Å).^14^ The model included the Cu_4_S_2_ core and 7 ligating His residues, where the α carbon and distal nitrogen were constrained at their crystallographic positions. A computational model for Cu*Z with a hydroxide bridging ligand and identical α carbon and distal nitrogen constraints was constructed from the crystal structure of *Paracoccus denitrificans* N_2_OR (*Pd*N_2_OR, PDB ID ; 1FWX).^13^ Calculations were performed using Gaussian 09 (version d01).^34^ Geometry optimizations were performed using the B3LYP functional, the TZVP basis set on all core atoms (Cu_4_S) and the ligating His nitrogens, and the SV basis set on all remaining atoms, and solvation was modeled with a PCM of 4.0. A larger basis set and different functionals were also explored, as described in the text. The optimized structures were then used for frequency, TD DFT, and single point calculations. To determine the relative energy of deprotonation (ΔΔ*E*) of the edge SH^–^ in the 2-hole *versus* 1-hole redox state, larger models were optimized that included two second sphere carboxylates, Asp127 and Asp240, which hydrogen bond to the His ligands of Cu_I_ and Cu_II_. The energy of an internal proton transfer from the edge SH^–^ to Asp127 was calculated for the 1-hole and 2-hole redox states and compared to obtain the ΔΔ*E*.

## 3 Results and analysis

### 3.1 Spectroscopy of 1-hole Cu_Z_

Previous spectroscopic studies of Cu_Z_, undertaken before identification of the presence of a second sulfur, were performed on samples of *Pp*N_2_OR and *Ps*N_2_OR that contained mixtures of the Cu_Z_ and Cu*Z sites (in a 7 : 3 ratio for *Pp*N_2_OR) without a way to resolve the spectral features of the Cu_Z_ site from the mixture.^19^,^28^,^29^ Recently, it has been found that the two-sulfur Cu_Z_ site cannot be reduced by methyl viologen, which reduces both the Cu_A_ site and the Cu*Z form of the cluster.^26^ This provides an opportunity to cleanly resolve the spectral features of 1-hole Cu_Z_ by studying methyl viologen reduced samples after removal of the reductant. This approach allows correlation of the electronic structure of 1-hole Cu_Z_, obtained from spectroscopy, with the recently determined Cu_4_S_2_ structure of the cluster, to determine the nature of the edge sulfur ligand in its 1-hole and resting 2-hole redox states.

#### EPR

**Fig. 2:**
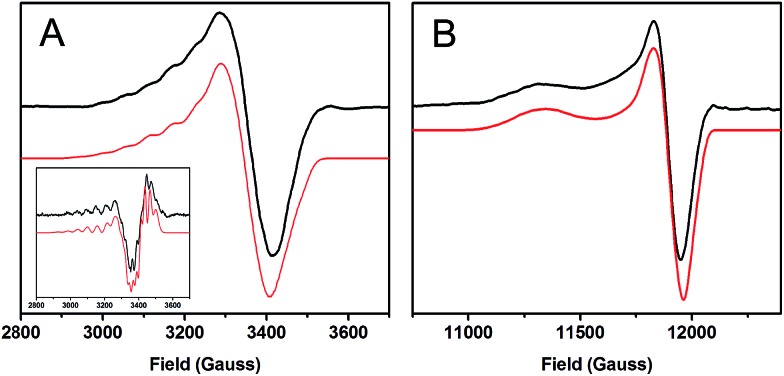
EPR spectra of 1-hole Cu_Z_ (black) with simulations (red). (A) X-band at 77 K, 9.6349 GHz. Inset: 2nd derivative of the X-band. (B) Q-band at 77 K, 34.082 GHz.

The X-band and Q-band EPR spectra of a methyl viologen reduced sample of 1-hole Cu_Z_ are given in Fig. 2. The EPR spectrum is axial with *g*_||_ > *g*_┴_ > 2.0 and a pattern of five evenly space hyperfine lines in the *A*_||_ region. The axial nature of the spectrum indicates that, while the spin density is delocalized over multiple copper nuclei, it resides in dominantly d_*x*^2^–*y*^2^_ orbitals on each Cu site that contributes to the ground state. The *A*_||_ hyperfine features can be further resolved in the second derivative of the X-band EPR spectrum, as can hyperfine features in the *A*_┴_ region (Fig. 2A inset and S2†). Simulation of the X-band, X-band 2nd derivative, and Q-band EPR spectra yields the *g* and *A* values for 1-hole Cu_Z_ given in Table 1. The *g* values for 1-hole Cu_Z_ are very similar to those previously obtained for 1-hole Cu*Z (Table 1) and to those obtained for Cu_Z_ in *Pp*N_2_OR.^15^,^29^ This is interesting, considering that an edge SH^–^ (thiolate) or S^2–^ (sulfide) in Cu_Z_ would be expected to be a more covalent ligand than the hydroxide in Cu*Z^15^ and this would lower the *g* values. However, in 1-hole Cu*Z there is a high energy d–d transition that is not present in the 1-hole Cu_Z_ spectrum (*vide infra*). This transition has previously been assigned as a d_*xy*_ → d_*x*^2^–*y*^2^_ excitation localized on Cu_I_.^35^ The *g*_||_ value is inversely proportional to the d_*xy*_ to d_*x*^2^–*y*^2^_ energy splitting, so the presence of a high energy d_*xy*_ → d_*x*^2^–*y*^2^_ transition in Cu*Z but not in Cu_Z_ would lead to a lower *g*_||_ value for Cu*Z than would be expected from covalency alone, which could result in similar *g*_||_ values between 1-hole Cu*Z and the more covalent 1-hole Cu_Z_ site. The ligand field origin of the lower energy d_*xy*_ → d_*x*^2^–*y*^2^_ transition in 1-hole Cu_Z_ is considered below.

**Table 1:** EPR *g* and *A* values for 1-hole Cu_Z_, obtained from simulations included in Fig. 2 and S2 with values for Cu*Z reproduced from ref. 15

	1-hole Cu_Z_	1-hole Cu*Z
*g*_||_	2.152	2.160
*A*_||_	56 × 10^–4^ cm^–1^	61 × 10^–4^ cm^–1^
	56 × 10^–4^ cm^–1^	23 × 10^–4^ cm^–1^
	56 × 10^–4^ cm^–1^	
*g*_┴_	2.042	2.043
*A*_┴_	20 × 10^–4^ cm^–1^	25 × 10^–4^ cm^–1^
	20 × 10^–4^ cm^–1^	20 × 10^–4^ cm^–1^
	20 × 10^–4^ cm^–1^	

The *A*_||_ and *A*_┴_ values for 1-hole Cu_Z_ are similar in magnitude to those for Cu*Z, but fitting the hyperfine pattern requires three equivalent contributions rather than the ∼5 : 2 ratio of hyperfine values observed for Cu*Z (Table 1).^36^ This indicates that in the ground state of 1-hole Cu_Z_ the spin is distributed over three copper centers in dominantly d_*x*^2^–*y*^2^_ orbitals. The three coppers involved are likely Cu_I_, Cu_II_, and Cu_IV_, since these copper centers are in the same plane as the two sulfur ligands and bonding with the strong donor μ_4_ sulfide and μ_2_ sulfur ligands should define a common *x*, *y* plane for these coppers, with the *z* axis of the local *g* tensor of each copper oriented perpendicular to the Cu_3_S_2_ plane. This is consistent with the axial nature of the *g* values and with the DFT calculations reported below.

#### Absorption and MCD

**Fig. 3:**
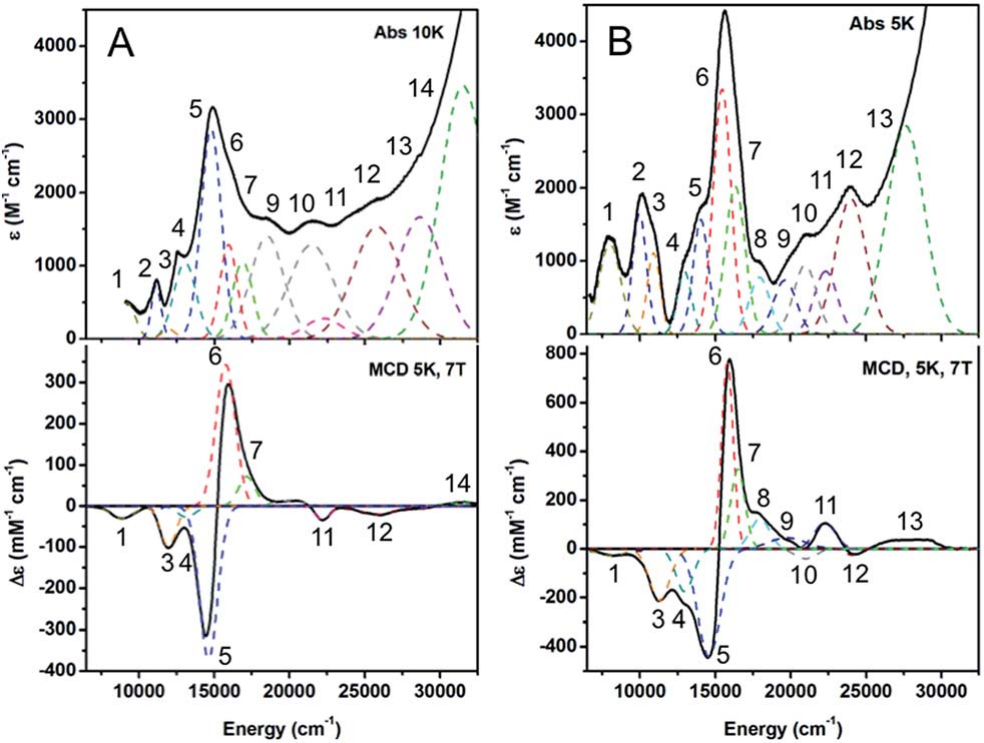
Low temperature absorption and MCD spectra of (A) 1-hole Cu_Z_, 10 K absorption, 5 K and 7 T MCD. (B) 1-hole Cu*Z, 5 K absorption, 5 K and 7 T MCD (adapted from ref. 15).

The low temperature absorption and MCD spectra of a methyl viologen reduced sample of 1-hole Cu_Z_ are presented in Fig. 3A. The absorption maximum of 1-hole Cu_Z_ occurs at 14 600 cm^–1^ (*ε* ≈ 3000 M^–1^ cm^–1^), 1000 cm^–1^ lower than the absorption maximum of 1-hole Cu*Z (Fig. 3B). There are no additional low energy intense absorption features due to the edge sulfur. The low temperature absorption and MCD spectra can be simultaneously fit to yield a total of 11 transitions, which can be assigned by considering their energies and *C*_0_/*D*_0_ ratios, following ref. 15 (Table S1†). Comparison of the transition assignments and energies of the 1-hole Cu_Z_ and 1-hole Cu*Z sites reveals some key differences. While the absorption maximum of Cu_Z_ occurs at lower energy than that of Cu*Z, from MCD the three μ_4_S to Cu CT transitions, assigned in Cu*Z,^35^ occur at very similar energies in the two sites (bands 5, 6, and 7, numbering given in Fig. 3). The shift in the absorption maximum therefore arises from a different intensity pattern for these transitions, where in Cu_Z_ the lowest energy transition at 14 600 cm^–1^ is the most intense (band 5) and the transition at 15 600 cm^–1^ is weaker (band 6), but in Cu*Z this is reversed. The μ_4_S^2–^ to Cu CTs in Cu*Z have previously been assigned as transitions from the three different 3p orbitals of the μ_4_S^2–^ to the β LUMO of the cluster. From our current study of resting 1-hole Cu*Z and Cu_Z_ with different edge ligands,^15^,^35^ the β LUMO is delocalized in the plane that contains Cu_I_, Cu_II_, Cu_IV_ and the μ_4_S^2–^, with different amounts of spin distributed over Cu_I_, Cu_II_, and Cu_IV_ depending on the edge ligation. Two of the μ_4_S^2–^ p orbitals are in the plane, oriented between Cu_I_ and Cu_IV_ (S p_*x*′_) and between Cu_IV_ and Cu_II_ (S p_*y*′_), while the third is perpendicular to the plane (S p_*z*′_). Scheme S1† reflects the orientation and simplified composition of these orbitals determined for 1-hole Cu*Z from DFT calculations. The CT intensities reflect the overlap of these three S p orbitals with the β LUMO. Since bands 5 and 6 show the highest intensity in the 1-hole forms of Cu_Z_ and Cu*Z, these must reflect charge transfer from the in-plane S p_*x*′_ (band 6, dominant in Cu*Z due to higher overlap with Cu_I_) and S p_*y*′_ (band 5) orbitals. Bands 6 and 5 form a pseudo-A feature in the MCD spectrum (*i.e.* derivative-shaped) and thus must arise from two transitions with orthogonal transition moments that spin–orbit couple in a third, mutually perpendicular direction (*i.e. L*_*z*_). Since band 6 arises from a transition to Cu_I_ (from its dominant intensity in Cu*Z), band 5 must reflect a transition to Cu_IV_, since the Cu_I_–S and Cu_IV_–S bonds are close to perpendicular (96° from crystallography) while the Cu_I_–S and Cu_II_–S bonds are close to parallel (160°).^13^ The change in relative intensities of the μ_4_S^2–^ to Cu CT transitions in Cu_Z_ relative to Cu*Z, where band 6 decreases in intensity while band 5 increases in intensity, thus indicates that there is less spin on Cu_I_ and more spin on Cu_IV_ in 1-hole Cu_Z_ relative to 1-hole Cu*Z. This is consistent with the EPR hyperfine values, which suggest that the spin in Cu_Z_ is delocalized 1 : 1 : 1 over Cu_I_, Cu_II_, and Cu_IV_, while from ref. 35 in Cu*Z the spin is delocalized ∼5 : 2 over Cu_I_ and Cu_IV_. Additionally, in Cu*Z, a band at 18 000 cm^–1^ (band 8) was assigned as a high energy d–d transition due to its high *C*_0_/*D*_0_ ratio; this was assigned as a localized d_*xy*_ → d_*x*^2^–*y*^2^_ transition on Cu_I_, where most of the 1-hole is localized.^35^ No equivalent high energy d–d transition is observed in the MCD spectrum of Cu_Z_. The lower energy of the d–d transitions in Cu_Z_ relative to Cu*Z is likely due to the decreased spin on Cu_I_, the only four coordinate site, relative to Cu_II_ and Cu_IV_, which are both 3 coordinate and have a weaker ligand field.

#### Resonance Raman

**Fig. 4:**
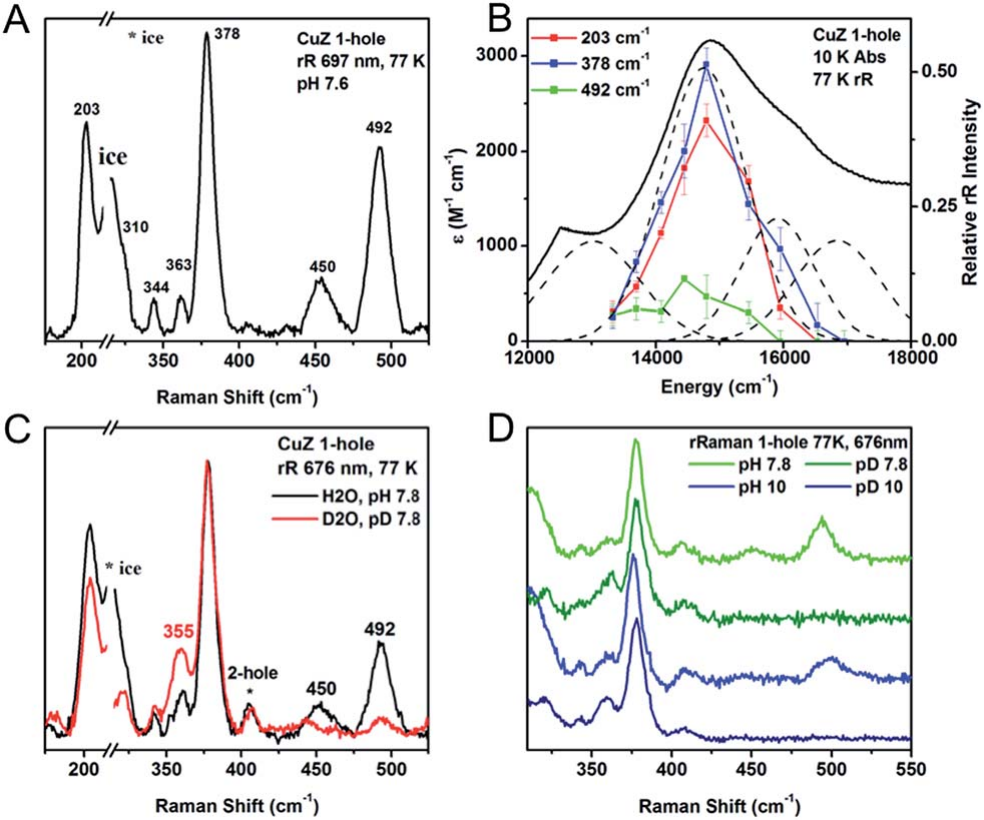
(A) Resonance Raman spectrum of 1-hole Cu_Z_ at 77 K, excitation energy 697 nm. (B) Excitation profile of the 203, 378, and 492 cm^–1^ vibrations. (C) H/D isotope shift of the vibrations of 1-hole Cu_Z_, performed in pH or pD 7.8, 100 mM phosphate, excitation energy 676 nm. (D) Comparison of SH bending vibrations at pH/pD 7.8 (green) and pH/pD 10 (blue).

The resonance Raman spectrum of 1-hole Cu_Z_ and the enhancement profiles of the vibrations are presented in Fig. 4A and B, respectively. Seven vibrations are enhanced in the most intense S to Cu CT transition (band 5), including three intense vibrations at 203, 378, and 492 cm^–1^. The vibration at 378 cm^–1^ occurs at the same energy as a Cu–S stretch of the Cu*Z site (Fig. S3†) and the previously reported ^34^S isotope sensitivities of both vibrations are similar (–5.8 and –4.7 cm^–1^, respectively),^28^ indicating that the 378 cm^–1^ vibration in 1-hole Cu_Z_ can be assigned as a Cu–S vibration of the μ_4_ sulfide. In contrast, the 203 cm^–1^ vibration is significantly lower in energy than the vibrations of Cu*Z, and thus can be assigned as a Cu–S vibration of the μ_2_ sulfur ligand that is only present in Cu_Z_. Further, there are two high energy vibrations in Cu_Z_ at 450 and 492 cm^–1^ that show significant deuterium isotope sensitivity, shifting down in energy by –137 cm^–1^ (for the 492 cm^–1^ vibration) in deuterated buffer (Fig. 4C). This shift requires their assignment as S–H bending modes. Thus, we can definitively identify the edge ligand in 1-hole Cu_Z_ as a μ_2_SH^–^. The S–H bending modes at 492 and 450 cm^–1^ are present at both pH 7.8 and pH 10 (Fig. 4D), indicating that the p*K*_a_ of the edge thiolate is ∼11 or higher. This is further supported by the lack of pH dependence observed in the MCD and EPR spectra of 1-hole Cu_Z_ between pD 6 and pD 10 (Fig. S4†). Since the second p*K*_a_ of free hydrogen sulfide in water is 12, a p*K*_a_ range of 11–12 can be estimated for the edge thiolate ligand in 1-hole Cu_Z_.

### 3.2 Spectroscopy of 2-hole Cu_Z_

#### Absorption

**Fig. 5:**
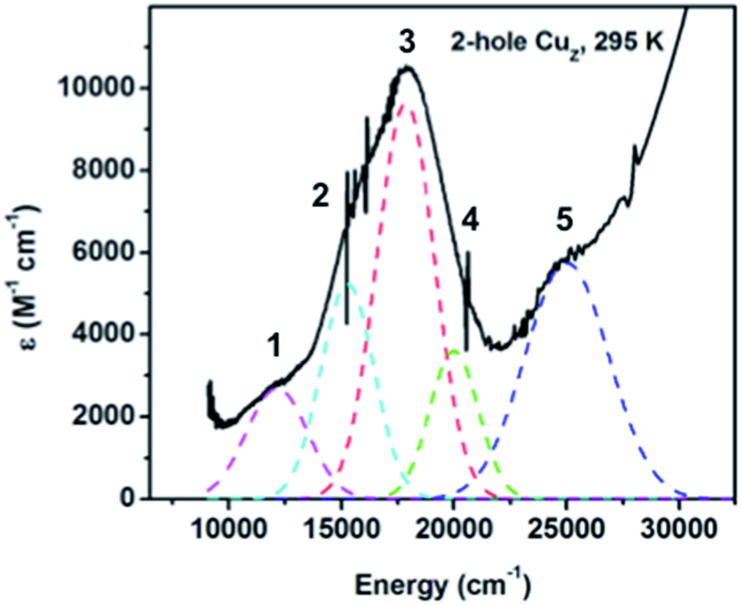
Absorption spectrum of 2-hole Cu_Z_ at room temperature, obtained after ascorbate reduction of Cu_A_ and subtraction of spectral contribution of 1-hole Cu*Z.

The 2-hole redox state has been previously shown to be the resting redox state of Cu_Z_. 2-hole Cu_Z_ is diamagnetic from MCD.^19^ The absorption features of 2-hole Cu_Z_ in as-isolated N_2_OR are present with additional spectral contributions from oxidized Cu_A_ and some amount of 1-hole Cu*Z.^26^ To remove these contributions, the absorption spectrum of 2-hole Cu_Z_ (Fig. 5) was obtained after reduction with sodium ascorbate, which reduces the Cu_A_ site faster than it reduces 2-hole Cu_Z_, and subtraction of the spectral contribution of 1-hole Cu*Z, obtained from a separately purified N_2_OR sample containing 90 ± 10% Cu*Z. An intense absorption maximum for 2-hole Cu_Z_ is observed at 18 300 cm^–1^ (*ε* ≈ 10 000 M^–1^ cm^–1^) with a weaker low energy shoulder, consistent with absorption spectra previously reported for ascorbate reduced samples containing high amounts of 2-hole Cu_Z_.^19^,^32^ The low temperature absorption spectrum of 2-hole Cu_Z_ (Fig. S5†) resolves this absorption maximum into three distinct absorption bands. Simulation of the room temperature absorption spectrum with transition energies derived from the low temperature spectrum distinguishes five transitions, all with absorption intensities higher than 1000 M^–1^ cm^–1^, sufficiently intense to be S to Cu CT transitions from the μ_4_S^2–^ or the μ_2_S ligand (Fig. 5). The two most intense transitions (bands 2 and 3) are either from different ligands (μ_2_S and μ_4_S^2–^) or from the same ligand to two different acceptor orbitals (the α and β holes of the broken symmetry singlet ground state). Based on the correlation of resonance Raman excitation profiles of the vibrations of 2-hole Cu_Z_ to DFT calculations (*vide infra*), the assignment of the two transitions as CT transitions from the μ_4_S^2–^ to two different holes is preferred.

#### Resonance Raman

**Fig. 6:**
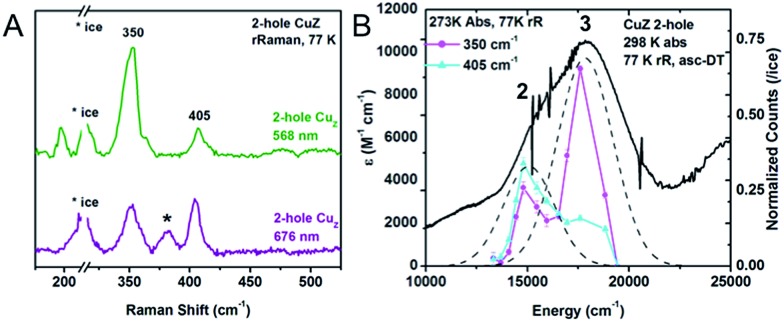
(A) Resonance Raman spectra of 2-hole Cu_Z_ at 77 K and two excitation energies, 568 nm (green) and 676 nm (purple). Starred vibration is due to 1-hole Cu*Z. (B) Excitation profiles of the 350 and 405 cm^–1^ vibrations overlaid with the room temperature absorption spectrum.

The resonance Raman spectrum of 2-hole Cu_Z_ was obtained upon excitation into the intense absorption maximum at 18 300 cm^–1^ (Fig. 6A). Two vibrations are enhanced at 350 and 405 cm^–1^. The ^34^S isotope shifts of these vibrations have been previously reported to be –5.6 and –5.8 cm^–1^, respectively, indicating that they are Cu–S stretches.^28^ In contrast to 1-hole Cu_Z_, no higher energy S–H bending vibration is observed (up to 800 cm^–1^). The excitation profile of the Cu–S stretching vibrations shows that they are enhanced differently in the most intense absorption bands 2 and 3 (Fig. 6B). The lower energy vibration at 350 cm^–1^ is enhanced in both transitions, while the higher energy vibration at 405 cm^–1^ is dominantly enhanced in the lower energy transition (band 2) and only weakly enhanced in band 3. This difference in profiling behavior is consistent with the Cu–S vibrations obtained computationally for a Cu_4_S_2_ cluster with a μ_2_S^2–^ and μ_4_S^2–^ and with the predicted enhancements of key vibrations in transitions from the μ_4_S^2–^ to the α and β holes (see 3.3).

The resonance Raman spectrum 2-hole Cu_Z_ shows no significant shift in the energies of the 350 and 405 cm^–1^ vibrations between pD 6 and pD 10 (Fig. S6†). This suggests that the edge ligand has a p*K*_a_ either lower than 5.5 or higher than 10.5. A p*K*_a_ higher than 10.5 in the 2-hole redox state is not consistent with observed p*K*_a_ of 11–12 for 1-hole Cu_Z_, as the increased charge of the 2-hole state will lead to a lower p*K*_a_ relative to the 1-hole redox state. The possibility of a p*K*_a_ less than 5.5 for 2-hole Cu_Z_ is evaluated computationally below.

### 3.3 Calculations

#### 1-hole Cu_Z_

**Fig. 7:**
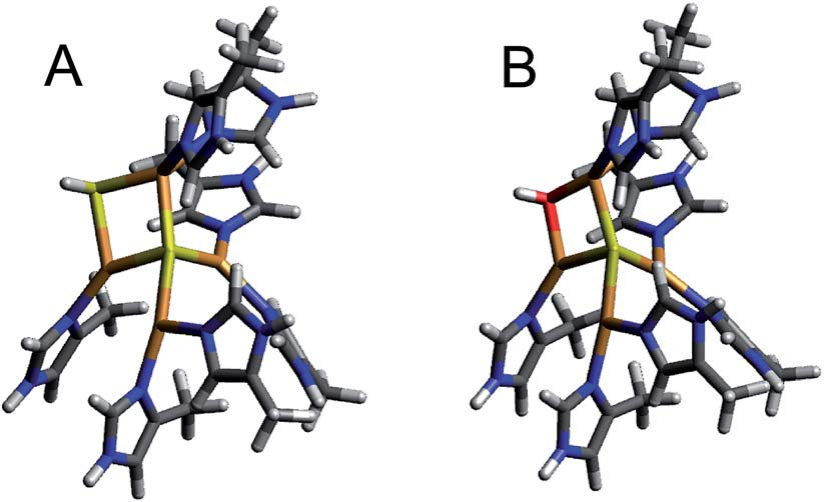
Computational models of (A) 1-hole Cu_Z_ and (B) 1-hole Cu*Z (B3LYP, TZVP on Cu, S, and ligating N atoms, and SV on all remaining atoms, PCM of 4.0).

A computational model of 1-hole Cu_Z_ was constructed based on the crystal structure of *Pseudomonas stutzeri* N_2_OR (PDB ID ; 3SBP, resolution 1.7 Å).^14^ On the basis of the resonance Raman data, the edge sulfur was modeled as an SH^–^ ligand bridging the Cu_I_–Cu_IV_ edge (Fig. 7A). This will be compared to an experimentally validated model of the Cu*Z site, which has a hydroxide ligand bridging the Cu_I_–Cu_IV_ edge (Fig. 7B).^15^ The optimized structure of the 1-hole SH^–^ cluster agrees well with the bond lengths and angles observed in the crystal structure (2.35 Å and 2.48 Å for the Cu_I_–μ_2_SH^–^ and Cu_IV_–μ_2_SH^–^ bonds computationally, relative to 2.61 Å and 2.49 Å crystallographically with a resolution of 1.7 Å,^14^ Table S2†). Since the crystal was grown from the “purple” resting form of *Ps*N_2_OR, containing the resting 2-hole redox state of the Cu_Z_ site, the Cu_Z_ site in the crystal may have some photo-reduction due to exposure to X-ray radiation.^37^ The calculated structures and spin distributions are not significantly perturbed when a triple zeta basis set is used on all His ring atoms (Tables S4 and S5†). Including the second sphere residues Lys397 and Glu435 in the computational model also does not affect the structure or spin distribution, consistent with the small effect on the spectral features of 1-hole Cu*Z observed experimentally upon deprotonation of Lys397.^15^ Thus, the structures including only first sphere ligands were used to model the Cu_Z_ and Cu*Z sites in this study.

The 1-hole model with an SH^–^ edge ligand reproduces the key spectral features observed for the 1-hole Cu_Z_ site. The Mulliken atomic spin distribution of the cluster with an SH^–^ edge ligand is delocalized over Cu_I_, Cu_II_, and Cu_IV_ in a 2 : 1 : 1 ratio. In going from Cu*Z to Cu_Z_ the calculated spin on Cu_I_ changes from 26% to 17% (Table 2), which is consistent with the decrease in intensity of band 6 observed in the absorption and MCD data for 1-hole Cu_Z_ and leads to a more equal distribution of spin over Cu_I_, Cu_II_, and Cu_IV_, consistent with the EPR hyperfine values. The LUMO of the Cu_Z_ model contains d_*x*^2^–*y*^2^_ character on Cu_I_, Cu_II_, and Cu_IV_, which are aligned, consistent with the ground state predicted from the EPR *g* values (Fig. S7†). It also contains significant antibonding μ_4_S^2–^and μ_2_SH^–^ character, explaining why Cu–S stretching vibrations of both the μ_4_S^2–^ and μ_2_SH^–^ are enhanced in the charge transfer transitions to this acceptor orbital. Additionally, the computational model predicts the Cu_Z_ site to be more covalent than the Cu*Z site, with 10% less Cu character in the ground state wavefunction, reflecting delocalization of the spin from Cu_I_ onto the edge SH^–^ ligand. The low g_||_ value for 1-hole Cu_Z_ is also predicted by the computational model (Table S7†). However, in contrast to experiment, the calculated *g*_||_ values for the Cu_Z_ and Cu*Z models differ, with a higher calculated *g*_||_ value for Cu*Z than that observed experimentally. This suggests that the calculated model of Cu*Z does not accurately predict the ligand field on Cu_I_ that leads to the higher energy d_*xy*_ → d_*x*^2^–*y*^2^_ transition observed experimentally.

**Table 2:** Mulliken atomic spin density of 1-hole computational models with SH^–^ and OH^–^ bridging ligands on the Cu_I_–Cu_IV_ edge, with % Cu and % d orbital character in their ground state wavefunctions

Edge ligand	Mulliken atomic spin density					
	Cu_I_	Cu_II_	Cu_III_	Cu_IV_	μ_4_S^2–^	μ_2_L^–^
SH^–^ bridge	0.17	0.11	0.06	0.10	0.34	0.16
OH^–^ bridge	0.26	0.09	0.04	0.13	0.31	0.10

The Cu–S stretching vibrations and S–H bending vibrations for the 1-hole SH^–^ model of Cu_Z_ are given in Table S8 and Fig. S8.† The model predicts two S–H bending modes at 426 and 461 cm^–1^ with H/D isotope shifts of –125 cm^–1^ and –123 cm^–1^, respectively, similar to the vibrations observed experimentally at 450 and 492 cm^–1^ (with a shift of –137 cm^–1^ for the 492 cm^–1^ vibration; the 450 cm^–1^ vibration cannot be observed after deuteration due to overlap with the ice scattering peak). Equivalent O–H bends are predicted for the OH bridged Cu*Z model at higher energies, but these are not experimentally observed. The Cu_Z_ model also predicts the presence of a low energy Cu–S stretching vibration of the μ_2_SH^–^ (178 cm^–1^, observed at 203 cm^–1^ experimentally) and both models show similar energies for the Cu–μ_4_S stretching vibrations. The absolute energies of the Cu–S stretching vibrations for both the μ_4_ sulfide and μ_2_ thiolate are underestimated, as has been found for computational models of the Cu*Z site.^15^,^35^ The TD DFT calculated absorption spectrum for the Cu_Z_ model is also very similar to the calculated absorption spectrum for the Cu*Z model both with B3LYP and with the functional B98, which has been shown to predict the experimental absorption spectrum of a Cu_3_S_2_ model complex reasonably well.^38^ Interestingly, neither the experimental absorption spectrum nor the TD-DFT calculation predicts an intense low energy charge transfer transition from the μ_2_SH^–^ ligand (Fig. S9†). While some weak transitions predicted computationally at lower energy than the μ_4_S^2–^ to Cu CT transitions have μ_2_SH^–^ to Cu CT character, they are predicted to lack intensity and are thus difficult to distinguish from the Cu d to d transitions that are also observed in this energy region.

Thus, a computational model of the tetranuclear copper cluster with an SH^–^ edge ligand bridging Cu_I_ and Cu_IV_ provides a good structural model of 1-hole Cu_Z_ that reproduces its key spectral features. This spectroscopically calibrated model was then extended to the 2-hole redox state of the Cu_Z_ site, for which less experimental data are accessible.

#### 2-hole Cu_Z_

**Fig. 8:**
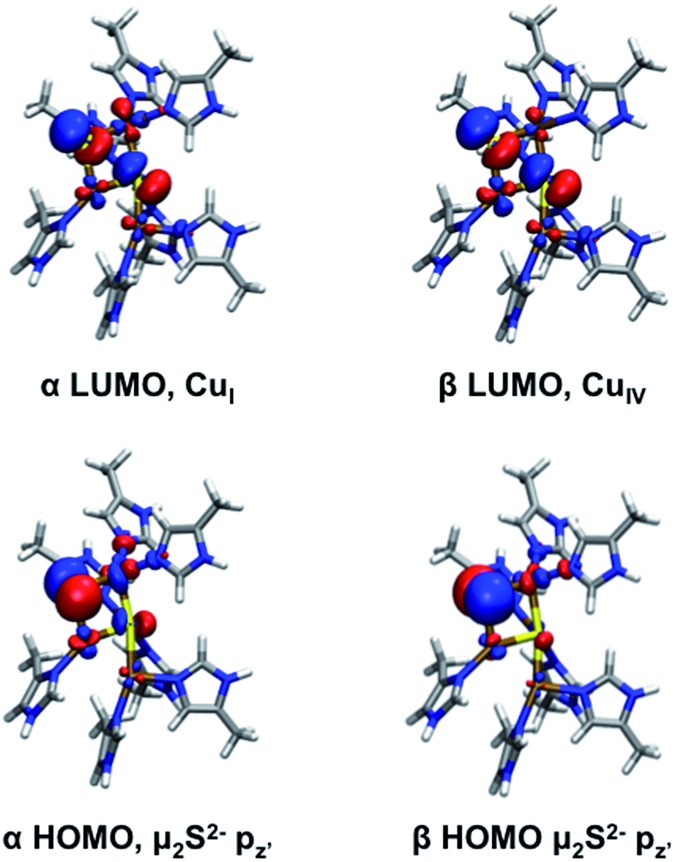
Frontier molecular orbitals of 2-hole Cu_Z_ (two sulfide model).

Two possible computational models were developed for 2-hole Cu_Z_, one with an edge thiolate ligand (Cu_4_S(SH)) and one with an edge sulfide (Cu_4_S_2_). These were optimized in both the triplet (*S* = 1) and broken symmetry singlet (*S* = 0) ground spin states. For both models the singlet is lower in electronic energy, by –8.0 kcal mol^–1^ for the sulfide and –3.4 kcal mol^–1^ for the thiolate (spin corrected energies using B3LYP). The singlet state was verified to be the ground state using a variety of functionals, including M06L, M06, and TPSSh. Thus, both structures would be consistent with the experimentally determined singlet ground state of 2-hole Cu_Z_.^19^ The optimized structure of the 2-hole Cu_4_S(SH) model is similar to that of the 1-hole SH^–^ model of 1-hole Cu_Z_, with slightly shorter Cu_I_/Cu_IV_–SH^–^ and Cu_I_/Cu_IV_–μ_4_S bonds (Table S9†). The α LUMO is dominantly localized on Cu_I_ and has equal μ_4_S^2–^ and μ_2_SH^–^ antibonding character (Table 3) while the β LUMO is delocalized equally over Cu_II_ and Cu_IV_ and has more μ_4_S^2–^ antibonding character. Upon deprotonation of the edge SH^–^, the 2-hole Cu_4_S_2_ model has significantly shorter bonds between the edge sulfide and Cu_I_/Cu_IV_ and similar μ_4_S^2–^–Cu bond lengths to the 2-hole SH^–^ model (Table S9†). In this model, the α LUMO is localized on Cu_I_ while the β LUMO is localized on Cu_IV_ (Fig. 8 and Table 3). Both holes have significant μ_2_S^2–^ character, indicating that the edge sulfide copper bonds are highly covalent.

**Table 3:** Mulliken spin densities on Cu and S atoms in the α and β LUMOs of the broken symmetry singlet 2-hole Cu_4_S(SH) and Cu_4_S_2_ models (B3LYP, TZVP on Cu, S, and ligating N atoms, and SV on all remaining atoms, PCM of 4.0)

		Mulliken spin density					
		μ_2_L	Cu_I_	Cu_II_	Cu_III_	Cu_IV_	μ_4_S^2–^
2-Hole SH^–^*S* = 0	α LUMO	0.16	0.37	0.06	0.06	0.04	0.20
	β LUMO	0.17	0.03	0.17	0.09	0.16	0.30
2-Hole S^2–^*S* = 0	α LUMO	0.33	0.22	0.06	0.04	0.07	0.23
	β LUMO	0.37	0.05	0.06	0.05	0.14	0.27

The energy of deprotonation calculated from the 2-hole models was compared to the calculated energy of deprotonation of the 1-hole SH^–^ model, where the experimentally estimated p*K*_a_ of the edge thiolate is 11–12 (*vide supra*). Examination of the energy required to deprotonate the 1-hole and 2-hole SH^–^ models shows that deprotonation of the 1-hole is not energetically favored (Δ*E* = 26 kcal mol^–1^, relative to an energy of –268 kcal mol^–1^ for a solvated proton)^39^ while deprotonation of the 2-hole is favorable (Δ*E* = –9 kcal mol^–1^). However, the two models have different charges (+2/+1 and +3/+2 for protonated and deprotonated 1-hole and 2-hole models, respectively) and this will significantly affect the relative energies of deprotonation. To minimize the charge effect, the computational models were expanded to include two second sphere Asp residues near the Cu_Z_ site, such that the 1-hole Cu_Z_ model with an edge thiolate is neutral and the 2-hole Cu_Z_ Cu_4_S(SH) model has a +1 charge. The proton transfer was performed internally to one of the Asp residues, so the total charge of the model does not change upon deprotonation (Fig. S10†). The ΔΔ*E* for deprotonation of the 2-hole edge thiolate relative to the 1-hole species is calculated to be –25 kcal mol^–1^ (with a dielectric of 4.0). This value is dependent on the dielectric (Fig. S11†) and, at high dielectric values, converges to a ΔΔ*E* of –12 kcal mol^–1^. To estimate the difference in p*K*_a_ between 1-hole and 2-hole Cu_Z_, the ΔΔ*G* was estimated from the ΔΔ*E* using frequency calculations for structures with identical fixed atom constraints and thus the same number and magnitude of imaginary frequencies (these Δ*G* corrections vary by only 0.3 kcal mol^–1^ between the protonated and deprotonated 1-hole structures). This gives a ΔΔ*G* of –12 kcal mol^–1^ for deprotonation of 2-hole *versus* 1-hole Cu_Z_, which corresponds to a Δp*K*_a_ of –9. Given the experimental p*K*_a_ value of 11–12 for the edge thiolate in 1-hole Cu_Z_, these calculations predict a p*K*_a_ for a thiolate in 2-hole Cu_Z_ of 3 or less, consistent with the absence of a pH effect in resonance Raman of 2-hole Cu_Z_ at pD 6. This strongly suggests that 2-hole Cu_Z_ is a two sulfide cluster at neutral pH.

The calculated spectral features for the Cu_4_S_2_ 2-hole Cu_Z_ model can be compared with those determined experimentally. The TD DFT predicted absorption spectrum (using both B3LYP and B98) is qualitatively similar to the experimental absorption spectrum, showing two intense absorption maxima with a higher energy shoulder (Fig. S12†). The predicted vibrations of the 2-hole Cu_4_S_2_ model are given in Table S10 and Fig. S13.† All of the calculated vibrations are shifted up in energy in comparison to those of the 1-hole SH^–^ model, with the most significant energy differences observed for the sulfur edge vibrations, due to the short and highly covalent Cu–μ_2_S^2–^ bonds in the 2-hole Cu_4_S_2_ cluster. In particular, the μ_2_S^2–^–Cu_I_ stretch now occurs at a similar energy to and mixes with vibrations of the μ_4_S^2–^, leading to symmetric and antisymmetric combinations of the μ_2_S^2–^–Cu_I_ and μ_4_S^2–^–Cu_I_ stretches (predicted at 312 and 309 cm^–1^, respectively, see ESI†). The symmetric combination is allowed in resonance Raman and will be enhanced in all transitions due to the high amount of μ_2_S^2–^ character in both the α and β holes. This is a good candidate for the 350 cm^–1^ vibration observed experimentally that profiles in both intense absorption bands (see Fig. 6B). The highest energy core vibration of the 2-hole μ_2_S^2–^ cluster is a symmetric Cu_II_–μ_4_S^2–^–Cu_IV_ stretch predicted at 344 cm^–1^ which will be selectively enhanced in a transition to the β LUMO localized on Cu_IV_ (Fig. 8). A symmetric Cu_II_–μ_4_S^2–^–Cu_IV_ stretch is also computationally predicted in the 1-hole SH^–^ model at 320 cm^–1^ and the calculated shift in energy of this mode between the 1-hole and 2-hole models (+24 cm^–1^) is similar to the energy increase of the highest energy Cu–S stretches observed experimentally in 1-hole and 2-hole Cu_Z_ (378 and 405 cm^–1^ respectively, Δ*ν* of +27 cm^–1^). Thus, the 2-hole Cu_4_S_2_ model qualitatively predicts a high energy Cu–S vibration that will be selectively enhanced only in a transition to the β hole and a lower energy Cu–S vibration that will be enhanced in both intense transitions. This is consistent with the enhancement profiles of the two vibrations observed experimentally in Fig. 6B. This establishes that a μ_2_S^2–^ bridge is energetically favored and consistent with the spectral features of 2-hole Cu_Z_.

## 4. Discussion

A combination of spectroscopic methods and DFT calculations has been used to define the protonation state of the μ_2_ sulfur ligand on the Cu_I_–Cu_IV_ edge in 1-hole and 2-hole Cu_Z_. This leads to insight into the spectroscopic similarities between 1-hole Cu_Z_ and 1-hole Cu*Z, the redox reactivity of 1-hole Cu_Z_ in the slow 2 electron reduction of N_2_O, and the interconversion between Cu_Z_ and Cu*Z, the reactive form of the cluster for N_2_O reduction *in vitro*.

### 4.1 Protonation states of 1-hole and 2-hole Cu_Z_

The protonation state of the edge ligand in 1-hole Cu_Z_ has been directly determined by resonance Raman spectroscopy. Two high energy vibrations are enhanced in the most intense μ_4_S^2–^ to Cu CT transition of 1-hole Cu_Z_, at 450 and 492 cm^–1^, and have large isotope shifts upon solvent deuteration (–137 cm^–1^ for the 492 cm^–1^ mode). This is consistent with S–H bending modes, indicating that the μ_2_S ligand is a thiolate. The energy and solvent isotope shift of these S–H bending modes are as predicted by DFT calculations for a model with a μ_2_SH^–^ bridging the Cu_I_–Cu_IV_ edge. The EPR spectrum of 1-hole Cu_Z_ indicates a ground state in which the spin is delocalized over 3 coppers in dominantly d_*x*^2^–*y*^2^_ orbitals. The absorption and MCD spectra show three μ_4_S^2–^ to Cu charge transfer transitions that have very similar energies to those observed for 1-hole Cu*Z (which has a hydroxide bridged Cu_I_–Cu_IV_ edge) but a different intensity pattern, consistent with a change in spin distribution in the cluster from dominantly on Cu_I_ in 1-hole Cu*Z to more evenly delocalized over Cu_I_, Cu_II_, and Cu_IV_. This ground state spin distribution is consistent with that predicted from DFT calculations for the μ_2_SH^–^. Based on the absence of a pH effect in 1-hole Cu_Z_ up to a pH of 10, the p*K*_a_ of the edge thiolate in 1-hole Cu_Z_ is estimated to be 11–12.

The 2-hole state of Cu_Z_ was also spectroscopically defined, but no direct spectroscopic evidence for the protonation state of the edge ligand was obtained. DFT calculations of the deprotonation of a μ_2_SH^–^ ligand in 2-hole Cu_Z_ relative to 1-hole Cu_Z_ were used to determine that 2-hole Cu_Z_ likely has a sulfide edge ligand. Deprotonation of a μ_2_SH^–^ ligand in the 2-hole redox state is at least 12 kcal mol^–1^ more favorable than in the 1-hole redox state, after accounting for charge and dielectric effects. This yields a calculated p*K*_a_ for a μ_2_SH^–^ ligand in 2-hole Cu_Z_ of 3 or less, which strongly suggests that 2-hole Cu_Z_ has an edge sulfide ligand at physiological pH. The calculated spectroscopic properties of a model of 2-hole Cu_Z_ with a μ_2_S^2–^ ligand are also consistent with those observed experimentally.

### 4.2 Similarities between 1-hole Cu_Z_ and 1-hole Cu*Z

It has previously been observed that the spectral features of 1-hole Cu_Z_ are rather similar to those of 1-hole Cu*Z, despite the change in the nature of the edge ligand from a thiolate to a hydroxide.^19^,^28^ The spectroscopic similarities between 1-hole Cu_Z_ and 1-hole Cu*Z reflect similar bonding interactions between the μ_4_S^2–^ and the in plane coppers (Cu_I_, Cu_II_, and Cu_IV_) which are not significantly perturbed by the nature of edge ligand. This results in similar transition energies in the absorption and MCD spectra, as the dominant transitions are due to μ_4_S^2–^ to Cu charge transfer, and a similar intense core Cu–μ_4_S^2–^ stretching mode in the resonance Raman spectrum, observed at 378 cm^–1^ in both sites. Small quantitative differences in the EPR hyperfine values and transition absorption and MCD intensities between the two sites arise from a perturbation of the spin density distribution of the cluster in 1-hole Cu_Z_, where the more covalent μ_2_SH^–^ leads to delocalization of the spin on Cu_I_ (dominant in Cu*Z) onto the edge SH^–^. Despite the higher covalency of the Cu_Z_ site, the *g* values in the EPR spectra are similar for Cu_Z_ and Cu*Z, as the localization of spin on the four coordinate Cu_I_ in 1-hole Cu*Z leads to higher energy d–d transitions, opposing the decreased covalency, leading to the net low *g* values also observed experimentally for Cu_Z_. The difference in edge ligation in the two sites is observed primarily in the resonance Raman enhanced vibrations, where a low energy Cu–μ_2_SH^–^ stretch at 203 cm^–1^ and higher energy S–H bending modes at 450 and 492 cm^–1^ are additionally enhanced in the dominant μ_4_S^2–^ to Cu CT transition in Cu_Z_ but not Cu*Z, due to the more covalent interaction between the coppers and the edge SH^–^. Thus, the spectral similarities between 1-hole Cu_Z_ and 1-hole Cu*Z reflect similar bonding with the μ_4_S^2–^ ligand and the distribution of spin over Cu_I_, Cu_II_, and Cu_IV_. The differences in the vibrational spectra of the two sites reflect the μ_2_SH^–^*versus* μ_2_OH^–^ edge ligation.

### 4.3 Insights into reactivity of 1-hole and 2-hole Cu_Z_

**Fig. 9:**
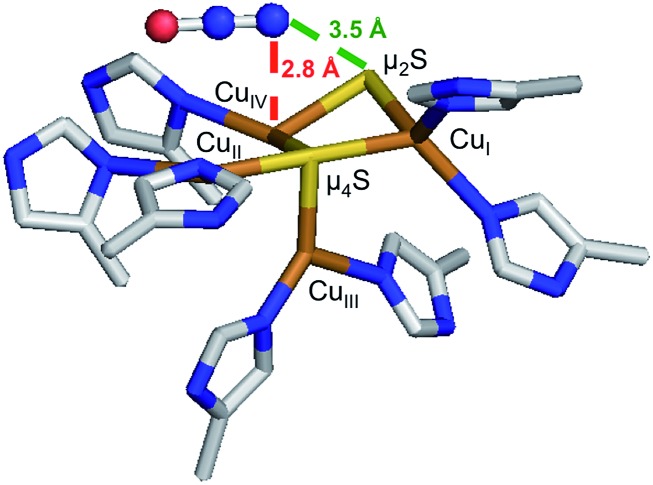
Crystal structure of N_2_O bound to Cu_Z_ (PDB ID: ; 3SBR, subunit C, resolution 1.7 Å).^14^

1-hole Cu_Z_ has been shown to perform a slow 2 electron reduction of N_2_O under single turnover conditions, with oxidation of both 1-hole Cu_Z_ and reduced Cu_A_ to generate resting 2-hole Cu_Z_ and 1 electron oxidized Cu_A_.^26^ A structure of *Ps*N_2_OR obtained from crystals pressurized with N_2_O shows a linear N_2_O molecule binding above the Cu_IV_–Cu_II_ edge of the Cu_Z_ cluster (Fig. 9).^14^ The O of N_2_O is thought to be oriented towards a solvent filled cavity between Cu_Z_ and Cu_A_, where there is a hydrogen bonding interaction with a localized solvent molecule, while the N end of the molecule is 2.8 Å from Cu_IV_ and 3.5 Å from the μ_2_SH^–^ ligand. The spectroscopically and computationally defined protonation states for 1-hole and 2-hole Cu_Z_ indicate that the 1-hole Cu_Z_ site will donate both an electron and a proton upon oxidation, due to the significantly decreased p*K*_a_ of the μ_2_SH^–^ in the 2-hole redox state. The participation of a proton in the reduction of N_2_O by 1-hole Cu_Z_ avoids the thermodynamically unfavorable 1-electron reduction of N_2_O to N_2_O^–^, which is endergonic by 25.4 kcal mol^–1^, while the proton-coupled reduction of N_2_O to form N_2_ and a hydroxyl radical is exergonic by 7.4 kcal mol^–1^.^40^ However, a substantial barrier exists for this process due to the fact that N_2_O is not activated through direct interaction with Cu_Z_ (the rate of N_2_O reduction by 1-hole Cu_Z_ is 2 × 10^–4^ s^–1^).^26^ Thus the N_2_O may alternatively be oriented with the O atom pointed towards Cu_Z_, where it can directly accept a proton and an electron from the μ_2_SH^–^ to break the N–O bond and generate resting 2-hole Cu_Z_, with transfer of the second electron from Cu_A_. Since no intermediate is observed in the reduction of N_2_O by 1-hole Cu_Z_,^26^ the hydroxide product that would be formed after N–O bond cleavage would likely be rapidly protonated and released into the nearby solvent-filled cavity, rather than coordinating to the Cu_Z_ cluster.

The 2-hole resting state of Cu_Z_ has been defined as having a highly covalent sulfide ligand bridging the Cu_I_–Cu_IV_ edge. This resting species is potentially the starting point for the chemical conversion of Cu_Z_ to Cu*Z, the reactive form of the cluster for N_2_O reduction.^26^*In vitro*, the presence of O_2_ is thought to promote the conversion of Cu_Z_ to Cu*Z, as isolation of N_2_OR in the presence of O_2_ results in samples with a high proportion of resting 1-hole Cu*Z, while the resting 2-hole state of Cu_Z_ is obtained when the purification is performed in the absence of oxygen.^16^ DFT calculations on the μ_2_S^2–^ model of 2-hole Cu_Z_ suggest that there are frontier molecular orbitals (FMOs) available to interact with O_2_. The α and β HOMOs of 2-hole Cu_Z_ are occupied μ_2_S^2–^ orbitals with dominant S p_*z*′_ character (50% and 66% μ_2_S^2–^ respectively, Fig. 8). This μ_2_S^2–^ p_*z*′_ orbital is oriented perpendicular to the Cu_3_S_2_ plane, towards the solvent-filled cavity where N_2_O, and by analogy O_2_, would access the Cu_Z_ cluster. Based on these FMOs, reaction of the Cu_Z_ site with O_2_ would proceed *via* oxidation of the edge sulfide, rather than by a Cu-based oxidation. Since this is a four electron process, there will in principle also be electrons available from the sulfide for the reduction of the copper site, dependent on the nature of the oxidized sulfur product. However, it is unlikely that this is the mechanism involved in interconversion of Cu_Z_ and Cu*Z*in vivo*, since resting Cu*Z has been isolated under exclusion of oxygen conditions from anaerobically grown cells in bacterial strains with accessory genes knocked-out.^41^ Thus, the *in vivo* mechanism for interconversion of Cu_Z_ and Cu*Z, which is required to maintain N_2_OR in the reactive Cu*Z form, and the role of accessory proteins in this process, remain to be identified.

## 5. Conclusions

We have used a combination of spectroscopies and DFT calculations to determine the protonation states of the edge sulfur in the 1-hole and 2-hole redox states of Cu_Z_. From resonance Raman spectroscopy, 1-hole Cu_Z_ has a μ_2_ thiolate ligand with a p*K*_a_ of 11–12, due to the presence of S–H bending modes that are not perturbed up to pH 10. DFT calculations of a 1-hole cluster with a μ_2_SH^–^ ligand reproduce the key spectral features of 1-hole Cu_Z_. The computational modeling of the 2-hole Cu_Z_ site indicates that the edge ligand is a μ_2_S^2–^ with a p*K*_a_ of 3 or less, which is consistent with the absorption and resonance Raman features of 2-hole Cu_Z_. The nature of this edge ligand has been used to obtain insight into the slow reduction of N_2_O by 1-hole Cu_Z_ and suggest how 2-hole Cu_Z_ might react with O_2_, a possible route for the conversion of Cu_Z_ to Cu*Z*in vitro*.

## Supplementary Material

Supplementary information
