# Supplementary material for: Protonation state of the Cu4S2 CuZ site in nitrous oxide reductase: redox dependence and insight into reactivity
Source: Chem Sci. 2015 Jul 3;6(10):5670–9. doi: 10.1039/c5sc02102b (PMC4583207; doi:10.1039/c5sc02102b)
Supplement: Supplementary file 1 [file SC-006-C5SC02102B-s001.pdf]

Electronic Supplementary Material (ESI) for Chemical Science.
This journal is © The Royal Society of Chemistry 2015

Electronic Supporting Information

Protonation state of the Cu_4S_2 Cu_Z site in nitrous oxide reductase: redox dependence and insight into reactivity

Esther M. Johnston, Simone Dell'Acqua, Sofia R. Pauleta, Isabel Moura and Edward I. Solomon

S1 Experimental Methodology

S1.1 Materials

All reagents were purchased from Sigma-Aldrich and of the highest grade commercially available. Deuterated water (99.9%), deuterated sodium hydroxide (99+%) and deuterated glycerol (99+% D) were obtained from Cambridge Isotopes.

Nitrous oxide reductase (N_2OR) was isolated from *Marinobacter hydrocarbonoclasticus* 617 (formerly *Pseudomonas nautica*) grown under microaerobic conditions in the presence of nitrate, as described previously.[1] MhN_2OR was isolated after two chromatographic steps that were performed aerobically, without added reductants, in Tris-HCl buffer at pH 7.6, as described by Dell'Acqua et. al.[1] These purification conditions were shown to maximize the amount of Cu_4S_2 Cu_Z content relative to Cu_4S Cu_Z* in the purified enzyme. Samples containing larger amounts of Cu_Z* were purified in parallel with three chromatographic purification steps from a batch of cells grown under anaerobic conditions in the presence of nitrate, and that had been stored at -80 ºC for a long period.[1, 2] The total MhN_2OR concentration was determined by bicinchoninic acid (BCA) assay with bovine serum albumin as the protein standard; copper content was determined by the 2,2'-biquinoline assay, as previously described.[1] MhN_2OR isolated by the first method had a copper content of 6.4 ± 0.2 Cu per monomer, while MhN_2OR isolated by the second method contained 6.2 ± 0.7 Cu per monomer, consistent with full occupancy of the copper sites in both the protein samples used. The total spin intensity quantified by EPR for the dithionite reduced MhN_2OR samples was one spin per monomer, consistent with the presence of Cu_Z or Cu_Z* in the 1-hole redox state and reduced Cu_A. The percentage of Cu_Z versus Cu_Z* in the samples used for this study was determined by EPR spin quantitation (Figure S1). The total spin of a dithionite reduced enzyme sample (where Cu_A and 2-hole Cu_Z are 1 electron reduced and only both 1-hole Cu_Z and 1-hole Cu_Z* contribute to the total spin)[2, 3] was compared to the total spin after 2 hours of reduction by 100 equivalents of reduced methyl viologen (which reduces 1-hole Cu_Z* to $4Cu^I$ but leaves 1-hole Cu_Z in the $3Cu^ICu^{II}$ state).[2] Samples purified with high amounts of Cu_Z contained 60±10% Cu_Z, while samples purified to obtain more Cu_Z* contained 10±10% Cu_Z. Purified MhN_2OR in 100 mM Tris-HCl buffer at pH 7.6 was stored frozen at -80ºC in small aliquots and thawed just prior to the preparation of spectroscopic samples.

S1.2 Spectroscopic studies

Spectroscopic samples of 1-hole and 2-hole Cu_Z were prepared in a glove box under N_2 atmosphere. Samples of 1-hole Cu_Z were prepared from *Mh*N_2OR (60% Cu_Z

and 40% Cu_Z*) that had been incubated with 100 equivalents of reduced methyl viologen. The methyl viologen was removed using a PD-10 Sephadex G-25 medium (GE HealthCare) desalting column with 100 mM phosphate at pH 7.6 as the elution buffer. The protein-containing column fractions were concentrated by centrifugation using Amicon Ultra concentrators with an Ultracell regenerated cellulose membrane (Millipore). For pH dependence studies, during the concentration step samples were buffer exchanged to 100 mM MES pD 6.0, 100 mM phosphate pD 7.6, or 100 mM CAPS pD 10. The total spin intensity observed by EPR was not changed by buffer exchanging to different pHs. To determine the effect of deuteration, samples were prepared in parallel at pH/pD 7.6 and pH/pD 10. Samples of 2-hole Cu_Z were prepared by reducing *Mh*N_2OR (60±10% Cu_Z, 40±10% Cu_Z*) with 10 equivalents of sodium ascorbate, which reduces the Cu_A site rapidly and the 2-hole Cu_Z site very slowly, and spectra were collected within 1 hour so that minimal reduction of 2-hole Cu_Z was observed. In parallel, MhN_2OR samples containing 90±10% Cu_Z* were reduced with 10 equivalents of sodium ascorbate to obtain the spectral features of 1-hole Cu_Z*. Corrected absorption spectra of 2-hole Cu_Z were obtained by subtracting the spectral contribution of the appropriate concentration of 1-hole Cu_Z*. Ascorbate reduced samples of *Mh*N_2OR containing Cu_Z were buffer exchanged by centrifugation to 100 mM MES pD 6.0, 100 mM phosphate pD 7.6, or 100 mM CAPS pD 10 for pH dependence experiments. Typical *Mh*N_2OR concentrations used for spectroscopic samples were 0.1-0.3 mM for absorption, MCD and EPR, and up to 0.5 mM for resonance Raman. The concentration of the dimer *Mh*N_2OR was determined using the extinction coefficient of 7100 M^{-1} cm^{-1} at 640 nm for the dimer in the dithionite reduced spectrum in the purified protein and corrected according to the volume changes involved in spectroscopic sample preparation.[4]

Absorption spectra were acquired in a Teflon-sealed 3 mm small volume quartz cell at room temperature using an Agilent 8453 UV-visible spectrophotometer with deuterium and tungsten sources. MCD samples were prepared by mixing protein samples in deuterated buffer 1:1 with deuterated glycerol. MCD spectra were collected on CD spectropolarimeters (Jasco J810 with an S20 PMT detector for the 300-900 nm region and a Jasco J730 with an InSb detector for the 600-2000 nm region) with sample compartments modified to insert magnetocryostats (Oxford Instruments SM4-7T). Low temperature absorption spectra were additionally obtained from the samples used for MCD using a double-beam Cary 500 spectrophotometer modified to accommodate a liquid helium cryostat (Janis Research Super Vari-Temp). Low temperature absorption spectra were corrected by subtracting the background spectrum from a cell containing a 50:50 mixture of buffer and deuterated glycerol and an additional scattering correction to account for the differences in glassing between the protein and background samples. EPR and resonance Raman samples were frozen in 4 mm diameter quartz sample tubes. EPR spectra were collected using a Bruker EMX spectrometer with an ER 041 XG microwave bridge, and an ER4102ST sample cavity for X-band and an ER 051 QR microwave bridge, an ER 5106QT resonator, and an Oxford continuous-flow CF935 cryostat for Q-band. X-band samples were run at 77 K in a liquid nitrogen finger dewar. Q-band samples were run at 77 K using a cooling He gas flow. EPR spectra were baseline corrected using WinEPR (Bruker) and simulated using Simfonia (Bruker). Resonance Raman spectra were collected using a series of lines from a Kr^+ ion laser (Coherent 190CK), a Ti-sapphire laser (M-squared SolsTice, pumped by a 12 W Lighthouse

Photonics Sprout diode pumped solid state laser), and a Dye laser (Rhodamine 6G, Coherent 699) with incident power of 20-30 mW arranged in a 130° backscattering configuration. The scattered light was dispersed through a triple monochromator (Spex 1877 CP, with 1200, 1800, and 2400 grooves mm^{-1} gratings) and detected with a back-illuminated CCD camera (Andor iDus model). Resonance Raman samples were immersed in a liquid nitrogen finger dewar at 77 K. The spectrum of black carbon in an identical quartz EPR tube was subtracted to remove the spectral contribution from quartz scattering. The intensity of the ice peak at ~229 cm^{-1} was used to normalize the intensities of vibrations to obtain resonance Raman excitation profiles.

S1.3 Computational Details

A computational model of Cu_Z was built from the atomic coordinates of the crystal structure of *Pseudomonas stutzeri* N_2OR, the only known structure of the Cu_4S_2 cluster (PDB ID 3SBP, resolution 1.7 Å).[5] The model included the Cu_4S_2 core and 7 ligating His residues, where the α carbon and distal nitrogen were constrained at their crystallographic positions. A computational model for Cu_Z* with a hydroxide bridging ligand and identical α carbon and distal nitrogen constraints was constructed from the crystal structure of *Paracoccus denitrificans* N_2OR (*Pd*N_2OR, PDB ID 1FWX).[6] Two larger structural models were also optimized: (1) including a second sphere Lys-Glu salt bridge near the Cu_I-Cu_{IV} edge in both sites (Lys397 for *Pd*N_2OR and Lys454 for *Ps*N_2OR)[7] for the 1-hole redox state of Cu_Z with an SH^- edge ligand and for 1-hole Cu_Z* with an OH^- edge ligand and (2) including two second sphere carboxylate residues, Asp127 and Asp240 (which hydrogen bond to the His ligands of Cu_I and Cu_{II}), in optimizations of 1-hole and 2-hole Cu_Z with SH^- or S^{2-} edge ligation. Including the second sphere residues did not significantly perturb the core Cu-S bond lengths (Table S3), geometries or the spin distribution of the cluster, so the smaller computational model lacking second sphere residues was used for the analysis of the spectroscopic properties of Cu_Z. Calculations were performed using Gaussian 09 (version d01).[8] Molecular structures and frequencies were visualized using Avogadro, an open source molecular builder and visualization tool (Version 1.1.1).[9] VMD 1.9.1 was used to visualize molecular orbitals,[10] and QMForge was used to obtain Mulliken spin populations of different orbitals and to analyze TD-DFT calculations.[11] Geometry optimizations were performed using the B3LYP functional, the TZVP basis set on all core atoms (Cu_4S_2) and the ligating His nitrogens, and the SV basis set on all remaining atoms. The optimizations of the large model (2), including two second sphere Asp residues, were additionally performed using a larger basis set with TZVP on the Cu_4S_2 core and all His ring heavy atoms. The resulting structures, spin distributions, and relative energies of the singlet and triplet ground states did not differ significantly from the smaller basis set optimizations (see Tables S4-S6), thus the structures optimized with TZVP only on the Cu_4S_2 core and ligating nitrogens were used for frequency, TD DFT, and single point calculations. Optimizations, single points, frequencies, and TD-DFT calculations were performed with PCM values of 4.0 and 10.0 and no significant change in the spin distribution, frequencies, or TD-DFT was observed. Spin distributions, frequencies, and TD-DFT results reported are from calculations with a PCM of 4.0. TD-DFT calculations were additionally performed with the functional B98, which has previously shown to predict the experimental spectrum of a Cu_3S_2 model complex.[12] As described in the Analysis,

models with an edge S^{2-} or SH^- were optimized for both 1-hole and 2-hole redox states and the 2-hole models were optimized in both triplet and broken symmetry singlet spin states (the singlet states were always lower in energy). This was further tested with the functionals M06L, M06, and TPSSh, which all predict that singlet ground states are lower in energy by at least 5 kcal/mol for the 2-hole redox state; however, these functionals predict a restricted singlet ground state wavefunction for the 2-hole Cu_Z site, while B3LYP predicts a more chemically reasonable spin polarized wavefunction.

To determine the relative energy of deprotonation (ΔΔE) of the edge SH^- in the 2-hole versus 1-hole redox state, the larger structural models including two second sphere Asp residues were used. The directly deprotonated versions of the 2Asp model (with loss of H^+ to solvent) were considered and the energies of an internal proton transfer from the edge SH^- to Asp127 were also calculated for the 1-hole and 2-hole redox states. The PCM dependence of the ΔΔE of deprotonation was also evaluated by obtaining the singlet point energies with different PCM values for structures optimized with a PCM of 4.0. The ΔG for deprotonation of 1-hole Cu_Z was also approximated from frequency calculations using structures with identical fixed atom constraints and the same number and magnitude for the imaginary frequencies associated with the fixed atom constraints. To minimize the error introduced into the vibrational energy correction by the fixed atom constraints, the masses of the fixed atoms were artificially increased until the calculated ΔG correction showed no further significant dependence on the fixed atom masses (~200 Da). The resulting ΔG was very similar to the calculated ΔE, indicating that the energy differences are electronic in origin.

S2 Supporting Figures, Tables, and Schemes

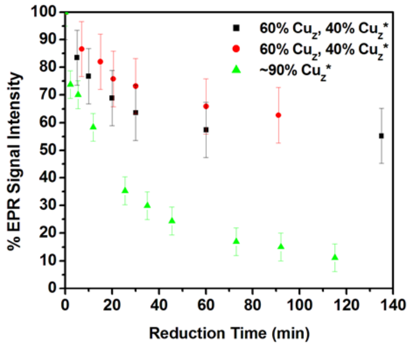

Figure S1: Methyl viologen reduction of three dithionite reduced samples of N_2OR to quantify the % Cu_Z from the % spin remaining after reduction. Black and red: N_2OR prepared with two aerobic chromatographic steps (“Form I”), containing ~60% Cu_Z and ~40% Cu_Z*. Green: N_2OR prepared with three aerobic chromatographic steps (“Form II”), containing ~90% Cu_Z* and ~10% Cu_Z.

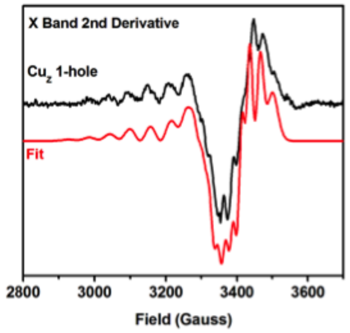

Figure S2: Second derivative (black) of the X band EPR spectrum of 1-hole Cu_Z at 77 K, 9.6349 GHz, with simulation in red.

	**1-hole Cu_Z**			**1-hole Cu_Z***		
Band	*Energy (cm^{-1})*	*C_0/D_0*	*Assignment*	*Energy (cm^{-1})*	*C_0/D_0*	*Assignment*
1	8900	-0.137	d-d	8000	-0.016	Cu_I dz^2
2	11400	---	IT	10000	----	IT
3	12000	-1.565	d-d	11100	-0.218	Cu_I dxz
4	13100	-0.054	d-d	12900	-0.194	Cu_I dyz
5	14600	-0.273	S $p_{y'}$	14300	-0.327	S $p_{y'}$
6	15800	0.565	S $p_{x'}$	15700	0.196	S $p_{x'}$
7	16900	0.144	S $p_{z'}$	16500	0.091	S $p_{z'}$
8				18000	0.170	Cu_I dxy
9	18500	---	His π_1	19800	-0.029	His π_1
10	21400	---	His π_1	21000	-0.045	His π_1
11	22200	-0.256	His π_1	22300	0.193	His π_1
12	25900	-0.027	His π_1	24000	-0.047	His π_1
13	28800	-0.005	His π_2	28100	-0.002	His π_2
14	31600	0.005	His π_2			

Table S1: Transition energies, C_0/D_0 ratios, and assignments from simultaneous fitting of the low temperature absorption and MCD spectra of 1-hole Cu_Z and 1-hole Cu_Z* (values and assignments for Cu_Z* reproduced from Ref. [7]).

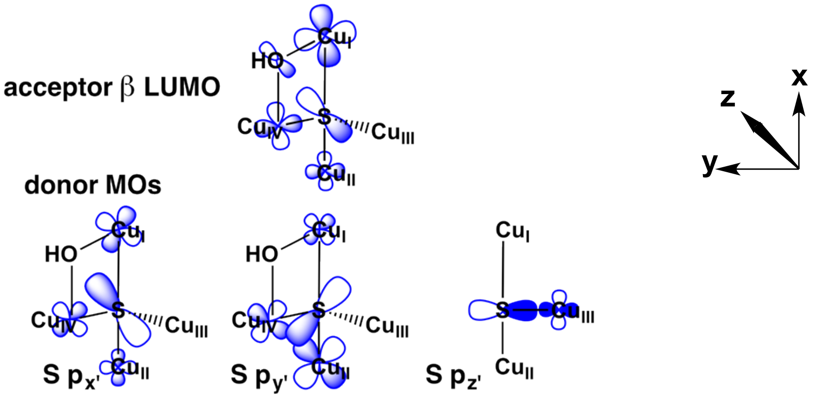

Scheme S1: Simplified acceptor and donor MOs for sulfide charge transfer transitions in resting Cu_Z*, derived from DFT calculations of the Cu_Z* cluster with a hydroxide bridged edge (B3LYP, tzvp on Cu_4SON_7, sv on remainder, PCM=4.0).

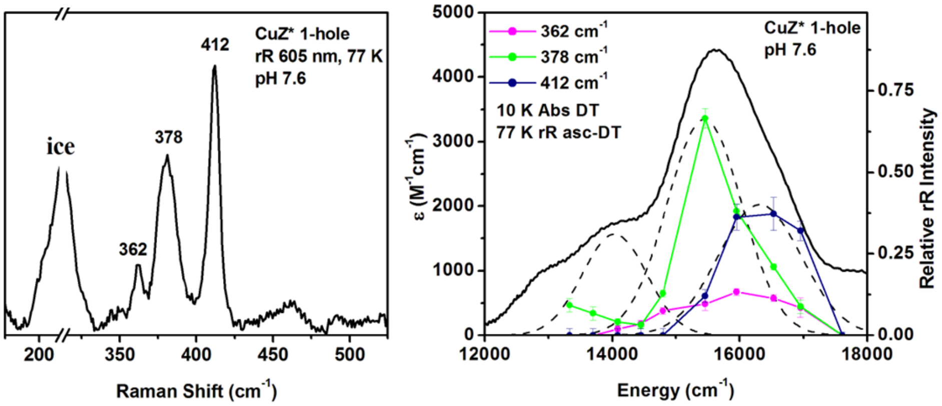

Figure S3: Resonance Raman spectrum of resting Cu_Z* at 77 K and 605 nm excitation; excitation profile of resting Cu_Z*. Reproduced from Ref. [7].

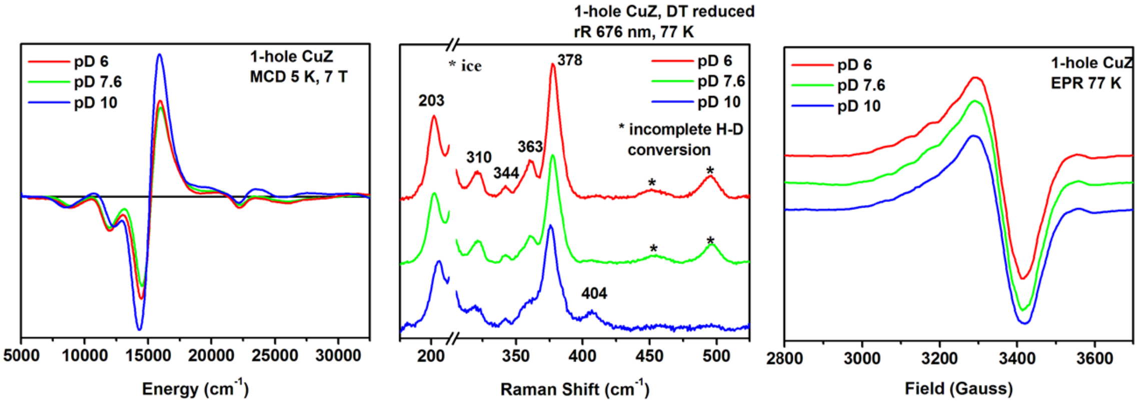

Figure S4: Lack of pH dependence of the MCD, resonance Raman, and EPR spectra of 1-hole Cu_Z at pD 6 (red), pD 7.6 (green) and pD 10 (blue). Note that the presence of weak S-H bends in the resonance Raman spectra at pD 6 and pD 7.6 is due to incomplete deuteration of the samples.

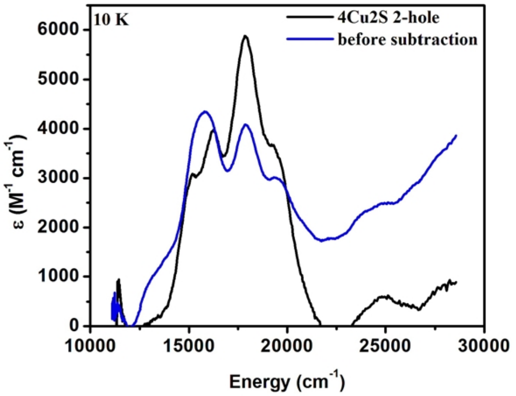

Figure S5: Absorption spectrum of 2-hole Cu_Z at 10 K, after ascorbate reduction and before (blue) or after (black) subtraction of the spectral contribution of 1-hole Cu_Z*.

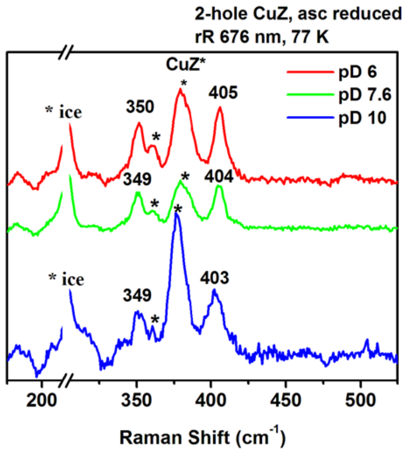

Figure S6: Resonance Raman spectra of ascorbate reduced N_2OR containing 2-hole Cu_Z and 1-hole Cu_Z* at pD 6.0 (red), pD 7.6 (green), and pD 10 (blue), with energies of the vibrations of the 2-hole Cu_Z site labeled.

Bond Lengths (Å)	PsN_2OR	1-hole SH	1-hole OH
Cu_I-S_1	2.443	2.437	2.431
Cu_{II}-S_1	2.187	2.264	2.258
Cu_{III}-S_1	2.223	2.229	2.240
Cu_{IV}-S_1	2.347	2.255	2.260
Cu_I-S_2	2.608	2.351	----
Cu_{IV}-S_2	2.493	2.478	----
Cu_I-OH	----	----	1.978
Cu_{IV}-OH	----	----	2.075
Cu_I-S_1-Cu_{IV}	89.5°	91.0°	80.6°
Cu_I-S_2-Cu_{IV}	82.8°	87.8°	----
S_1-S_2	3.538	3.379	----
Cu_I-Cu_{IV}	3.374	3.350	3.037
Cu_I-Cu_{III}	3.435	3.320	3.670
Cu_{II}-Cu_{IV}	2.822	2.694	2.732
Cu_{II}-Cu_{III}	2.763	3.162	2.814
Cu_{III}-Cu_{IV}	2.951	3.343	3.037

Table S2: Comparison of the important bond lengths and angles for the Cu_Z site obtained from X-ray crystallography of *Ps*N_2OR (PDB ID 3SBP, resolution 1.7 Å) and the optimized structure of the 1-hole Cu_Z site with an SH^- edge ligand (B3LYP, TZVP on Cu, S, and ligating N atoms, and SV on all remaining atoms, PCM of 4.0). For comparison, the optimized structure of the OH bridged model of resting Cu_Z* is also included.

Bond Lengths (Å)	1-hole SH		1-hole S^{2-}		2-hole SH^-		2-hole S^{2-}	
		2Asp		2Asp		2Asp		2Asp
Cu_I-S_1	2.437	2.464	2.641	2.565	2.385	2.422	2.383	2.406
Cu_{II}-S_1	2.264	2.262	2.256	2.258	2.270	2.269	2.288	2.285
Cu_{III}-S_1	2.229	2.226	2.240	2.229	2.232	2.229	2.229	2.225
Cu_{IV}-S_1	2.255	2.244	2.263	2.267	2.217	2.215	2.202	2.194
Cu_I-S_2	2.351	2.350	2.194	2.216	2.340	2.338	2.229	2.215
Cu_{IV}-S_2	2.478	2.508	2.305	2.319	2.448	2.470	2.278	2.293
S_1-S_2	3.379	3.402	3.461	3.443	3.164	3.206	3.010	3.074
Cu_I-N_{24}	2.098	2.057	2.015	2.021	2.030	1.997	2.066	2.018
Cu_I-N_{30}	2.198	2.270	3.403	3.028	2.100	2.149	2.215	2.394
Cu_{II}-N_{18}	2.116	2.085	2.193	2.156	2.043	2.022	2.095	2.071
Cu_{II}-N_4	2.060	2.051	2.069	2.055	2.045	2.033	2.058	2.046
Cu_{III}-N_{12}	2.026	2.028	2.050	2.048	1.998	2.003	2.009	2.013
Cu_{III}-N_{36}	2.076	2.088	2.089	2.111	2.051	2.061	2.066	2.073
Cu_{IV}-N_{50}	2.037	2.034	2.074	2.086	1.993	1.995	2.001	2.006

Table S3: Comparisons of key bond lengths for small computational models (white columns) of 1-hole and 2-hole Cu_Z and large computational models (grey columns) including two second sphere Asp residues (optimized with B3LYP; tzvp $Cu_4S_2N_7$/sv; PCM=4.0).

Bond Lengths (Å)	1-hole SH 2Asp		1-hole S^{2-} 2Asp		2-hole SH^- 2Asp		2-hole S^{2-} 2Asp	
Cu_I-S_1	2.464	2.488	2.565	2.580	2.422	2.438	2.406	2.420
Cu_{II}-S_1	2.262	2.262	2.258	2.257	2.269	2.275	2.285	2.289
Cu_{III}-S_1	2.226	2.227	2.229	2.231	2.229	2.229	2.225	2.228
Cu_{IV}-S_1	2.244	2.245	2.267	2.269	2.215	2.215	2.194	2.190
Cu_I-S_2	2.350	2.346	2.216	2.216	2.338	2.332	2.215	2.204
Cu_{IV}-S_2	2.508	2.499	2.319	2.313	2.470	2.468	2.293	2.289
S_1-S_2	3.402	3.392	3.443	3.433	3.206	3.187	3.074	3.053
Cu_I-N_{24}	2.057	2.068	2.021	2.031	1.997	2.007	2.018	2.017
Cu_I-N_{30}	2.270	2.295	3.028	3.032	2.149	2.171	2.394	2.484
Cu_{II}-N_{18}	2.085	2.092	2.156	2.167	2.022	2.027	2.071	2.074
Cu_{II}-N_4	2.051	2.057	2.055	2.059	2.033	2.039	2.046	2.051
Cu_{III}-N_{12}	2.028	2.039	2.048	2.060	2.003	2.010	2.013	2.024
Cu_{III}-N_{36}	2.088	2.090	2.111	2.114	2.061	2.064	2.073	2.074
Cu_{IV}-N_{50}	2.034	2.042	2.086	2.097	1.995	1.998	2.006	2.006

Table S4: Basis set dependence of the calculated structures of 1-hole and 2-hole Cu_Z for large computational models (white: tzvp $Cu_4S_2N_7$/sv; grey: tzvp $Cu_4S_2His_7$/sv, B3LYP, PCM=4.0.

Model	Mulliken Atomic Spin Density					
	Cu_I	Cu_{II}	Cu_{III}	Cu_{IV}	μ_4S^{2-}	μ_2S
1-hole SH^- 2Asp	0.17	0.11	0.05	0.10	0.34	0.16
	0.14	0.13	0.05	0.11	0.35	0.16
1-hole S^{2-} 2Asp	0.15	0.04	0.01	0.09	0.27	0.40
	0.11	0.04	0.02	0.16	0.29	0.34

Table S5: Basis set dependence of the Mulliken atomic spin density distribution of 1-hole Cu_Z with 2 Asp residues (white: tzvp $Cu_4S_2N_7$/sv; grey: tzvp $Cu_4S_2His_7$/sv, B3LYP, PCM=4.0).

		Mulliken Atomic Spin Density					
		Cu_I	Cu_{II}	Cu_{III}	Cu_{IV}	μ_4S^{2-}	μ_2S
2-hole SH^- S=0	α LUMO	0.37	0.06	0.06	0.04	0.20	0.16
	β LUMO	0.03	0.17	0.09	0.16	0.30	0.17
	α LUMO	0.36	0.06	0.06	0.04	0.21	0.16
	β LUMO	0.03	0.21	0.07	0.15	0.29	0.14
2-hole S^{2-} S=0	α LUMO	0.22	0.06	0.04	0.07	0.23	0.33
	β LUMO	0.05	0.06	0.05	0.14	0.27	0.37
	α LUMO	0.18	0.07	0.04	0.08	0.25	0.34
	β LUMO	0.07	0.08	0.05	0.14	0.28	0.33

Table S6: Basis set dependence of the Mulliken atomic spin density in the α and β LUMOs of 2-hole Cu_4S_2 and 2-hole $Cu_4S(SH)$ (white: tzvp $Cu_4S_2N_7$/sv, grey: tzvp $Cu_4S_2His_7$/sv, B3LYP and PCM=4.0).

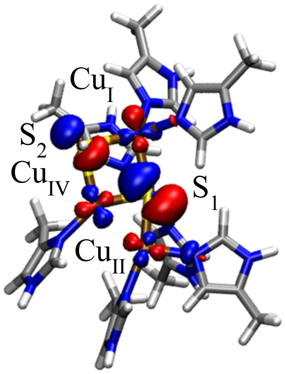

Figure S7: Ground state wavefunction of the 1-hole SH model of Cu_Z.

	g_x	g_y	g_z
**SH bridge**	2.044	2.057	2.158
**OH bridge**	2.055	2.076	2.243

Table S7: Computationally predicted g values for SH^- bridged model of 1-hole Cu_Z (B3LYP, tzvp on $Cu_4S_2N_7$, sv on remainder, PCM=4.0) and OH^- bridged model of 1-hole Cu_Z* with second sphere Lys and Glu (B3LYP, tzvp on Cu_4SN_7O, sv on remainder, PCM=4.0), calculated using Orca.[13]

**1-hole SH^- bridge**		**1-hole OH bridge**	
Energy (cm^{-1}) (H/D shift)	*Vibration*	*Energy (cm^{-1}) (H/D shift)*	*Vibration*
461 (-125)	S-H bend ‖ to Cu_I-Cu_{IV}	682 (-175)	O-H bend ‖ to Cu_I-Cu_{IV}
426 (-123)	S-H bend ⊥ to Cu_I-Cu_{IV}	510 (-128)	O-H bend ⊥ to Cu_I-Cu_{IV}
		396 (-8)	Cu_I-OH
320 (+1)	Cu_{II}-μ_4S-Cu_{IV} sym	341 (-1)	Cu_{II}-S-Cu_{III}-Cu_{IV}
310 (+6)	Cu_{III}-μ_4S	312 (-3)	Cu_{III}-S-Cu_{IV} sym
299 (-2)	Cu_I-μ_4S-Cu_{IV} sym	285 (0)	Cu_{III}-S-Cu_{IV} antisym
		268 (-3)	Cu_{IV}-OH
242 (-2)	Cu_I-μ_2S		
178 (0)	Cu_{IV}-μ_2S		
162 (0)	Cu_I-μ_4S	209 (0)	Cu_I-S

Table S8: Vibrations predicted for the 1-hole SH^- bridged model of Cu_Z and the 1-hole OH bridged model of Cu_Z*. Stretching vibrations of the μ_2 edge ligand are highlighted in grey.

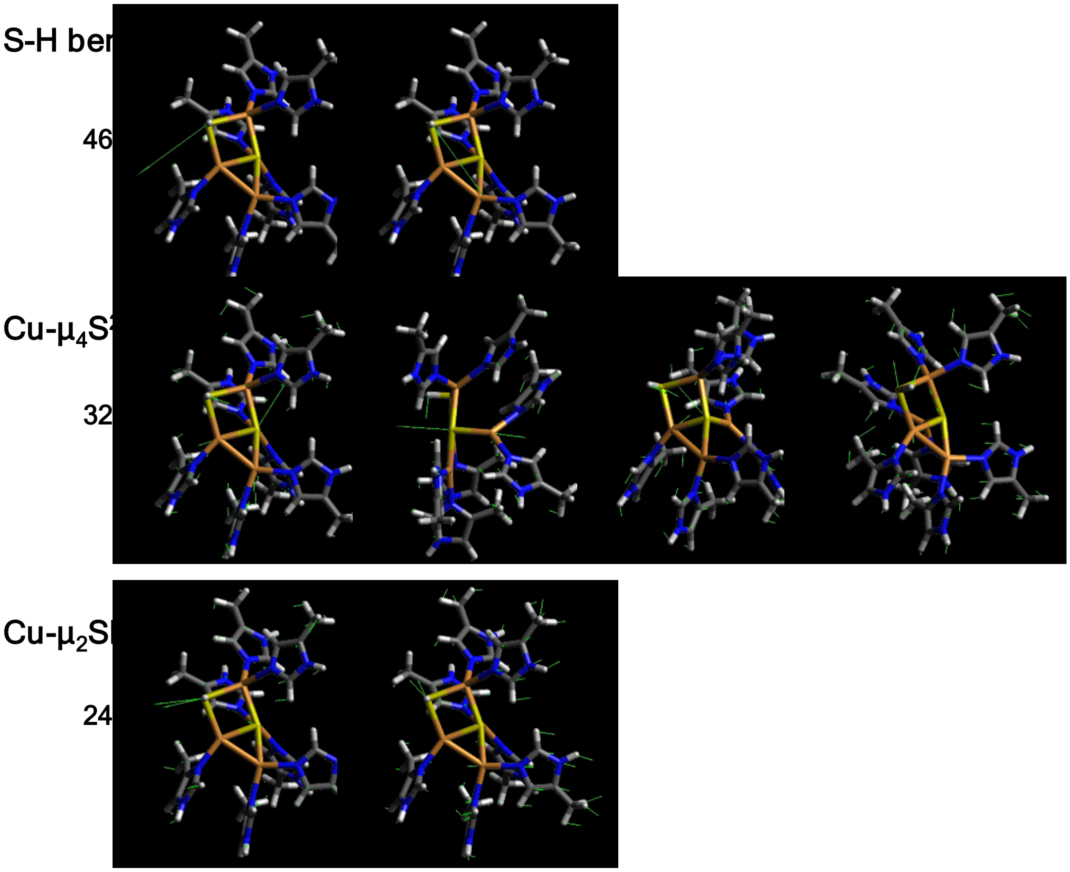

Figure S8: Pictorial representations of the vibrations of the 1-hole SH^- model listed in Table S4.

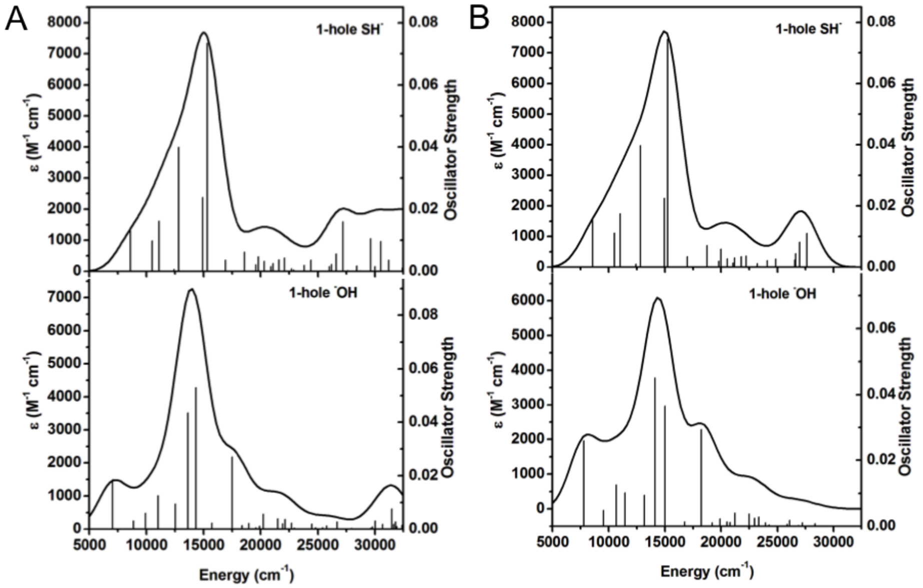

Figure S9: TD DFT predicted absorption spectra of the 1-hole SH^- bridged model of Cu_Z (top) and the OH bridged model of Cu_Z* (bottom) with two functionals: A) B3LYP, and B) B98.

Bond Lengths (Å)	1-hole SH	2-hole SH$^-$	2-hole S^{2-}
Cu_I-S_1	2.437	2.385	2.383
Cu_{II}-S_1	2.264	2.270	2.288
Cu_{III}-S_1	2.229	2.232	2.229
Cu_{IV}-S_1	2.255	2.217	2.202
Cu_I-S_2	2.351	2.340	2.229
Cu_{IV}-S_2	2.478	2.448	2.278
Cu_I-S_1-Cu_{IV}	91.0°	97.5	95.8°
Cu_I-S_2-Cu_{IV}	87.8°	92.6	98.0°
S_1-S_2	3.379	3.164	3.010
Cu_I-Cu_{IV}	3.350	3.463	3.403
Cu_I-Cu_{III}	3.320	3.177	3.341
Cu_{II}-Cu_{IV}	2.694	2.660	2.682
Cu_{II}-Cu_{III}	3.162	3.208	3.164
Cu_{III}-Cu_{IV}	3.343	3.317	3.383

Table S9: Comparison of important bond lengths and angles of the broken symmetry singlet 2-hole 4CuS(SH) and 4Cu2S models with the computational model of 1-hole Cu_Z (B3LYP, TZVP on Cu, S, and ligating N atoms, and SV on all remaining atoms, PCM of 4.0).

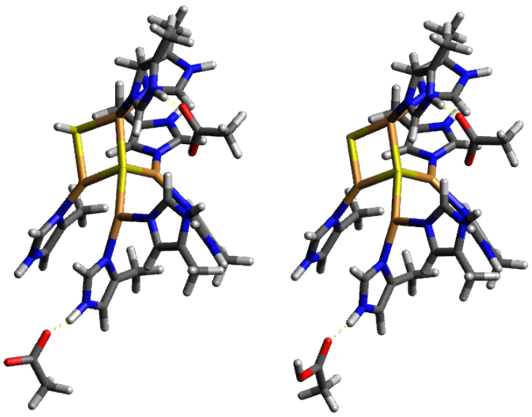

Figure S10: Structural models of 2-hole SH$^-$ with second sphere Asp residues and 2-hole S^{2-} model after internal proton transfer.

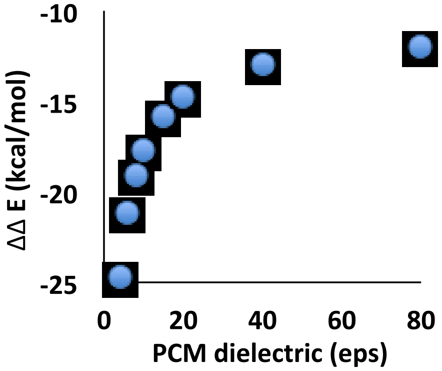

Figure S11: Dependence of the ΔΔE for deprotonation of the 2-hole 4CuS(SH) model (+1 charge) relative to the 1-hole 4CuS(SH) model (neutral) on the dielectric used for the PCM.

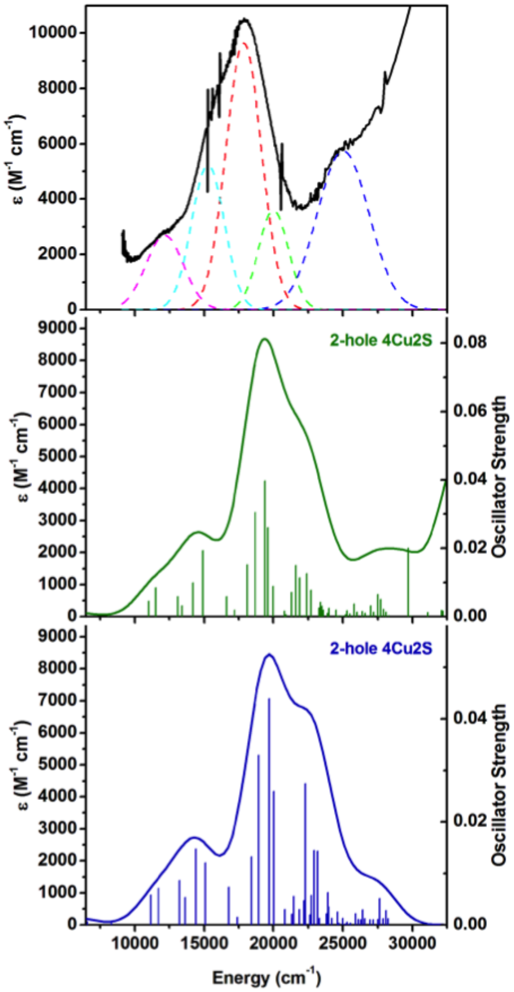

Figure S12: Comparison of the experimental absorption spectrum of 2-hole Cu_Z (black) and the TD DFT predicted absorption spectrum of the broken symmetry singlet 2-hole 4Cu2S model (green, B3LYP; blue, B98; TZVP on $Cu_4S_2N_7$, SV on remainder, PCM of 4.0).

2-hole S^{2-} (S=0)		1-hole SH^- (S=1/2)	
Energy (cm^{-1})	*Vibration*	*Energy (cm^{-1})*	*Vibration*
		461	S-H bend // to Cu_I-Cu_{IV}
		426	S-H bend ⊥ to Cu_I-Cu_{IV}
344	Cu_{II}-μ_4S-Cu_{IV} sym	320	Cu_{II}-μ_4S-Cu_{IV} sym
312	μ_2S-Cu_I-μ_4S sym, Cu_{III}-μ_2S	310	Cu_{III}-μ_4S
309	μ_2S-Cu_I-μ_4S antisym, Cu_{III}-μ_2S		
297	Cu_{II}-μ_4S	299	Cu_I-μ_4S-Cu_{IV} sym
		242	Cu_I-μ_2S
256	Cu_{IV}-μ_2S	178	Cu_{IV}-μ_2S
202	Cu_I-μ_4S	162 (0)	Cu_I-μ_4S

Table S10: Predicted vibrations of the 2-hole 4Cu2S model of 2-hole Cu_Z and the 1-hole SH^- model of 1-hole Cu_Z, vibrations with edge ligand character highlighted in grey.

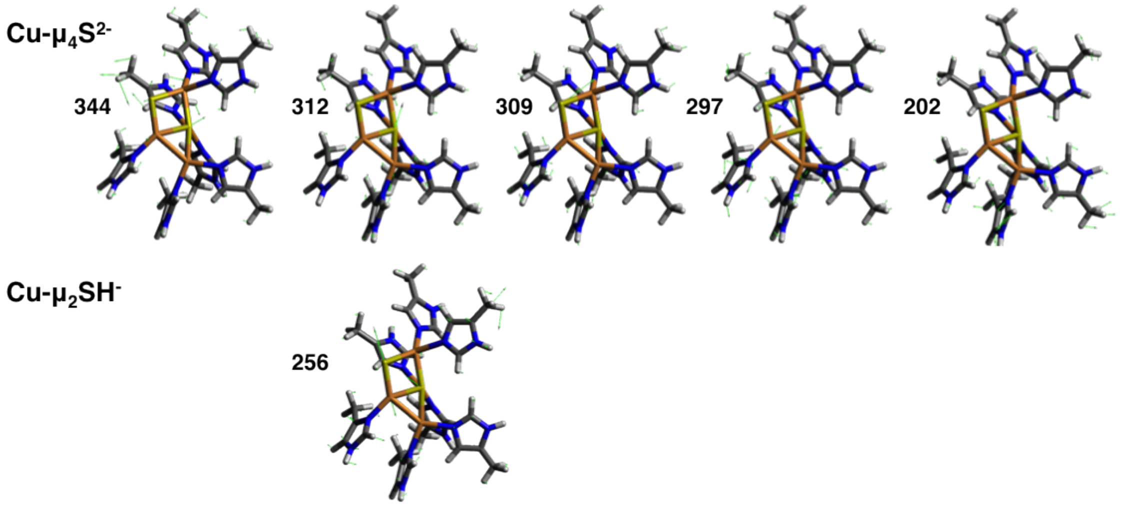

Figure S13: Pictorial representations of the vibrations of the 2-hole μ_2S^{2-} model listed in Table S6.

Calculated Structures

1-hole SH^- small model (B3LYP; tzvp $Cu_4S_2N_7$/sv; PCM=4.0)
2 2

Cu	15.86139	27.55597	1.79755
Cu	12.74928	26.73854	0.98044
Cu	13.70871	25.35849	-1.6976
Cu	15.23553	24.5845	0.38261
S	16.63083	25.38456	2.26727
S	14.67052	26.70118	-0.14984
N	10.8479	26.8082	0.28351
N	9.04306	26.5699	-0.97603
N	12.66796	22.11305	-4.17502
N	12.93728	23.70057	-2.64538
N	14.65004	20.62702	-0.93098
N	14.93705	22.59769	0.04673
N	12.44895	26.55091	3.02619
N	12.20806	27.04802	5.17805
N	14.94305	28.69998	3.4348
N	13.757	30.195	4.58299
N	16.54144	29.32417	0.89527
N	16.71898	31.01201	-0.53001
N	13.24926	26.94095	-3.02532
N	12.54999	28.77303	-4.08699
C	10.34988	26.98969	-0.93461
C	9.82961	26.26193	1.05574
C	8.69711	26.10499	0.28674
C	7.36497	25.56102	0.61601
C	13.47622	23.11861	-3.71994
C	11.56026	22.04478	-3.34597
C	11.72884	23.03508	-2.39655
C	10.79505	23.40098	-1.29493
C	15.32373	21.81892	-0.96343
C	13.79875	20.64682	0.16634
C	13.98108	21.87646	0.76882
C	13.32991	22.439	1.98394
C	11.82032	27.34213	3.89135
C	13.26121	25.71403	3.78026
C	13.12532	26.00638	5.11981
C	13.79395	25.449	6.31296
C	13.96169	29.59036	3.36096
C	15.40285	28.7359	4.74617
C	14.68279	29.66452	5.4767
C	14.78501	30.121	6.88399
C	16.40314	29.6851	-0.37487
C	16.97783	30.45348	1.58238
C	17.0973	31.51697	0.70766
C	17.51601	32.92799	0.886
C	13.30511	28.27144	-3.0407
C	12.44548	26.56758	-4.08474

C	11.99951	27.68529	-4.74351
C	11.08802	27.77498	-5.87499
H	10.88161	27.39413	-1.78978
H	9.97489	26.00718	2.10065
H	8.42887	26.61196	-1.78401
H	7.12842	24.65164	0.02913
H	6.55523	26.29201	0.42317
H	7.31589	25.28673	1.68287
H	12.85194	21.52202	-4.98047
H	14.41766	23.38857	-4.18987
H	10.76188	21.32278	-3.48977
H	10.24597	24.33375	-1.51614
H	10.04939	22.60305	-1.137
H	11.33412	23.55946	-0.34386
H	14.78235	19.85165	-1.57312
H	16.06243	22.06972	-1.71892
H	13.15676	19.8108	0.4273
H	12.81391	23.39317	1.76507
H	14.07055	22.63672	2.7816
H	12.5812	21.73684	2.38929
H	11.10021	28.11761	3.64694
H	13.90857	24.97271	3.32554
H	11.86807	27.49935	6.02185
H	14.41799	24.58341	6.0351
H	14.45847	26.19372	6.79481
H	13.06967	25.10933	7.07839
H	13.38485	29.82945	2.47221
H	16.21586	28.09948	5.08543
H	13.08263	30.92682	4.78462
H	15.07255	31.18895	6.95236
H	13.83008	30.00597	7.43293
H	15.55129	29.53901	7.42317
H	16.08648	29.03521	-1.18492
H	17.17388	30.43528	2.65007
H	16.71566	31.52797	-1.40477
H	18.40918	33.17443	0.27902
H	16.71651	33.63885	0.59867
H	17.77111	33.12412	1.94102
H	13.84112	28.89416	-2.33062
H	12.22151	25.5313	-4.31285
H	12.42667	29.75271	-4.32515
H	10.78863	26.76741	-6.20869
H	10.16036	28.33038	-5.62946
H	11.53842	28.28677	-6.74902
H	17.75519	25.40603	1.51642

1-hole OH^- small model (B3LYP; tzvp $Cu_4S_2N_7$/sv; PCM=4.0)
2 2
C 21.7600010000 43.4680050000 13.8479930000
C 22.4559810000 42.8500230000 15.0341560000
N 22.2048690000 41.5400730000 15.4668000000

C 23.3939490000 43.4104860000 15.8871700000
C 22.9611830000 41.3273530000 16.5441940000
N 23.7059930000 42.4349990000 16.8339960000
H 21.6526130000 42.7383410000 13.0311300000
H 20.7468740000 43.8189100000 14.1137020000
H 23.8527010000 44.3950900000 15.8944730000
H 22.9972460000 40.4113790000 17.1265760000
H 24.3522700000 42.5388250000 17.6106340000
C 18.5340020000 45.9070030000 11.4620000000
C 18.5089860000 44.5723460000 12.1217350000
N 18.0959990000 44.3620020000 13.4380020000
C 18.8667520000 43.3216870000 11.6569870000
C 18.2180670000 43.0241370000 13.7199370000
N 18.6857510000 42.3725370000 12.6593190000
H 17.5320890000 46.3780880000 11.4399890000
H 18.8799770000 45.8079680000 10.4198690000
H 19.2459580000 43.0505430000 10.6764420000
H 17.9757520000 42.5878140000 14.6847510000
H 17.7561890000 45.0755710000 14.0764140000
C 17.4769980000 42.6500030000 18.5280000000
C 18.0630370000 41.6145090000 17.6525410000
N 17.3850030000 40.4789930000 17.2110080000
C 19.3181590000 41.5290890000 17.0887700000
C 18.2371410000 39.7638490000 16.4122910000
N 19.4089400000 40.3800810000 16.3193460000
H 16.5770110000 43.1180070000 18.0821070000
H 17.1733210000 42.2441770000 19.5133610000
H 20.1471950000 42.2234400000 17.1889390000
H 17.9621210000 38.8434270000 15.9097110000
H 16.4330120000 40.2210810000 17.4543510000
C 14.7850100000 33.8560010000 13.5820050000
C 16.0263110000 34.6953190000 13.5586720000
N 16.4239850000 35.4900000000 14.6369940000
C 17.0095700000 34.8927470000 12.6031460000
C 17.6030150000 36.1015570000 14.3184900000
N 17.9778100000 35.7735730000 13.0882980000
H 14.8252270000 33.0881520000 14.3788540000
H 14.6630610000 33.3326770000 12.6190670000
H 17.0765320000 34.4627370000 11.6082710000
H 18.1426740000 36.7647820000 14.9855960000
H 15.9390800000 35.5601940000 15.5268420000
C 16.3110060000 36.1230050000 7.1490040000
C 16.7896110000 36.5968780000 8.4763030000
N 16.0009990000 37.3419970000 9.3540050000
C 17.9973480000 36.4327780000 9.1339590000
C 16.7450950000 37.5817260000 10.4874900000
N 17.9545200000 37.0492700000 10.3771630000
H 16.0067580000 36.9599630000 6.4899950000
H 17.1102730000 35.5651460000 6.6329870000
H 18.8852790000 35.9116750000 8.7846580000
H 16.3648540000 38.1300010000 11.3448350000

H 15.0377050000 37.6240220000 9.1978130000
C 20.5880130000 38.8279890000 7.1139770000
C 19.9508840000 39.3873800000 8.3430080000
N 18.6039740000 39.7310010000 8.4179900000
C 20.4584410000 39.7087580000 9.5869020000
C 18.3522920000 40.2605390000 9.6512640000
N 19.4581520000 40.2557860000 10.3846940000
H 20.5461620000 39.5401190000 6.2666650000
H 21.6486650000 38.5979010000 7.3087310000
H 21.4670370000 39.5528770000 9.9503600000
H 17.3769570000 40.6254060000 9.9591840000
H 17.9279940000 39.6317610000 7.6670290000
C 23.2859940000 41.3879980000 11.0910050000
C 23.9278860000 40.8259470000 12.3379070000
N 23.5407210000 39.6053000000 12.9152740000
C 24.9465260000 41.3591170000 13.1105800000
C 24.3072490000 39.4177130000 13.9900230000
N 25.1660320000 40.4670240000 14.1610390000
H 23.6854070000 42.3935330000 10.8757540000
H 22.1901820000 41.4681760000 11.2042750000
H 25.5165760000 42.2767310000 12.9990350000
H 24.2608570000 38.5668120000 14.6642590000
H 25.8736980000 40.5532010000 14.8836300000
Cu 19.4323900000 36.8271170000 12.0371950000
Cu 20.8419820000 40.1815390000 14.8129040000
Cu 19.3419920000 40.4724330000 12.4496800000
Cu 21.7551700000 38.6523660000 12.7409130000
S 19.6906700000 38.6195970000 13.6587630000
O 21.3177420000 37.0593780000 11.4848300000
H 13.8774300000 34.4666030000 13.7528010000
H 18.2119590000 43.4504540000 18.7148950000
H 15.4385470000 35.4463710000 7.2404260000
H 20.0964230000 37.8911600000 6.7873600000
H 19.2170370000 46.6104350000 11.9770200000
H 22.3310730000 44.3352340000 13.4743740000
H 23.4881100000 40.7475890000 10.2124460000
H 21.8434620000 36.3065520000 11.7773800000

2-hole SH^- small model, S=0 (B3LYP; tzvp $Cu_4S_2N_7$/sv; PCM=4.0)
3 1
Cu 15.65823 27.6691 1.76304
Cu 12.71994 26.73202 1.00224
Cu 13.67199 25.38976 -1.7519
Cu 15.13829 24.56938 0.31009
S 16.42529 25.49663 2.17437
S 14.63163 26.67881 -0.14801
N 10.86237 26.74981 0.26729
N 9.04303 26.56999 -0.97598
N 12.66798 22.1131 -4.1748
N 12.92966 23.71812 -2.66591

N	14.64999	20.62699	-0.931
N	14.88984	22.61465	0.00911
N	12.45192	26.55184	3.02778
N	12.20805	27.048	5.178
N	14.91333	28.68303	3.44487
N	13.75701	30.19498	4.58298
N	16.26094	29.4106	0.91155
N	16.71891	31.01203	-0.53007
N	13.18331	26.95353	-2.973
N	12.55002	28.77302	-4.08693
C	10.35241	26.97255	-0.94179
C	9.84675	26.19506	1.03856
C	8.70442	26.0773	0.27821
C	7.36497	25.561	0.616
C	13.46777	23.13181	-3.74207
C	11.56972	22.0385	-3.33535
C	11.73015	23.03996	-2.39734
C	10.79497	23.4009	-1.29518
C	15.28617	21.83291	-0.99861
C	13.81914	20.63496	0.18141
C	13.97005	21.87766	0.763
C	13.33002	22.43901	1.984
C	11.82306	27.34716	3.89329
C	13.2546	25.70624	3.78464
C	13.11952	26.0025	5.12274
C	13.79396	25.44901	6.31297
C	13.94218	29.59062	3.3666
C	15.38147	28.71118	4.75373
C	14.67593	29.65485	5.47957
C	14.78501	30.12099	6.88399
C	16.28589	29.72726	-0.37961
C	16.70913	30.53016	1.61332
C	17.00374	31.54335	0.72083
C	17.51601	32.92798	0.886
C	13.28317	28.28487	-3.02466
C	12.37101	26.57334	-4.02729
C	11.96963	27.6871	-4.71963
C	11.08807	27.77501	-5.87496
H	10.87409	27.40063	-1.79015
H	9.99723	25.91193	2.07512
H	8.41702	26.6477	-1.77291
H	7.08371	24.69058	-0.00801
H	6.57709	26.32741	0.47908
H	7.33239	25.23752	1.6693
H	12.85021	21.51652	-4.97735
H	14.3999	23.40961	-4.22592
H	10.77958	21.30489	-3.46479
H	10.24189	24.3311	-1.5157
H	10.0522	22.60013	-1.14234
H	11.32988	23.55388	-0.34016
H	14.79657	19.84305	-1.56071

H	16.00453	22.09087	-1.77067
H	13.21065	19.78219	0.46689
H	12.793	23.38338	1.76934
H	14.07684	22.6436	2.77402
H	12.59578	21.72944	2.4015
H	11.10413	28.12369	3.6494
H	13.88392	24.94567	3.33917
H	11.85958	27.49391	6.02222
H	14.40875	24.57601	6.0382
H	14.46563	26.19402	6.78366
H	13.0734	25.12166	7.08631
H	13.36838	29.83922	2.47931
H	16.18711	28.06534	5.09139
H	13.092	30.9355	4.78751
H	15.0989	31.18162	6.94018
H	13.82454	30.03576	7.42673
H	15.5341	29.52566	7.43179
H	16.02564	29.07325	-1.20544
H	16.7967	30.53756	2.69452
H	16.84709	31.49349	-1.41634
H	18.48593	33.06843	0.37169
H	16.8118	33.6779	0.47704
H	17.66894	33.1585	1.95304
H	13.83849	28.91408	-2.33661
H	12.11695	25.53968	-4.2302
H	12.46179	29.74958	-4.35484
H	11.58618	28.22798	-6.75503
H	10.74471	26.77154	-6.17578
H	10.18697	28.38732	-5.67346
H	17.52601	25.61015	1.39473

2-hole S^{2-} small model, S=0 (B3LYP; tzvp $Cu_4S_2N_7$/sv; PCM=4.0)
2 1

Cu	15.23096	24.54935	0.33875
Cu	13.69156	25.36458	-1.69996
Cu	12.72744	26.7311	0.9859
Cu	15.86465	27.57147	1.76888
S	14.68315	26.64056	-0.07963
S	16.51355	25.4504	1.99241
N	12.54991	28.77318	-4.08708
N	13.19784	26.95621	-2.97113
N	16.71899	31.01205	-0.53007
N	16.43966	29.3618	0.9126
N	13.75697	30.19496	4.58298
N	14.94252	28.69924	3.43732
N	12.20789	27.04799	5.17804
N	12.45695	26.56105	3.02715
N	14.91995	22.59702	0.0319
N	14.65012	20.62699	-0.93089
N	12.93552	23.70174	-2.64707
N	12.66792	22.11308	-4.17514

N	9.04337	26.56951	-0.97597
N	10.84714	26.80972	0.28202
C	11.08806	27.77497	-5.875
C	11.97491	27.68422	-4.72005
C	12.38747	26.57393	-4.02609
C	13.28722	28.28486	-3.02417
C	17.51602	32.92796	0.88603
C	17.05995	31.5301	0.71226
C	16.87644	30.48831	1.60001
C	16.36265	29.69905	-0.36809
C	14.78504	30.121	6.88397
C	14.68257	29.66443	5.47722
C	15.40228	28.73477	4.74763
C	13.96085	29.58894	3.36234
C	13.79403	25.449	6.31295
C	13.13251	26.01241	5.11786
C	13.27666	25.72799	3.77759
C	11.81775	27.34344	3.89363
C	13.32961	22.43902	1.9837
C	13.97423	21.87485	0.76551
C	13.80258	20.64127	0.16947
C	15.31348	21.81949	-0.97485
C	10.79524	23.4011	-1.29457
C	11.72825	23.03453	-2.39658
C	11.56107	22.04323	-3.34506
C	13.4748	23.1203	-3.72212
C	7.36485	25.56119	0.61606
C	8.69775	26.10366	0.28668
C	9.83006	26.26128	1.05555
C	10.34807	26.99185	-0.93595
H	11.58121	28.23556	-6.75421
H	10.18324	28.38033	-5.66729
H	10.74994	26.77085	-6.18066
H	12.45435	29.74916	-4.35258
H	12.14106	25.5374	-4.22699
H	13.83722	28.91795	-2.33419
H	17.70765	33.14153	1.95094
H	16.7643	33.65893	0.52895
H	18.45583	33.12808	0.33492
H	16.76636	31.51246	-1.41272
H	17.03037	30.4828	2.67438
H	16.07333	29.04137	-1.18197
H	15.55258	29.5401	7.42247
H	13.83073	30.00422	7.4337
H	15.07093	31.18939	6.95249
H	13.081	30.92512	4.78527
H	16.21413	28.09681	5.08683
H	13.38432	29.82837	2.47361
H	13.0651	25.07745	7.05924
H	14.4328	26.19931	6.82032
H	14.44271	24.60381	6.02913

H	11.8598	27.49027	6.02333
H	13.96091	25.02466	3.31543
H	11.09018	28.11267	3.65152
H	12.59394	21.73068	2.40185
H	14.07736	22.65344	2.76973
H	12.80116	23.38644	1.76515
H	13.1721	19.79849	0.43624
H	16.04945	22.07264	-1.73186
H	14.78856	19.84731	-1.56672
H	11.3341	23.55731	-0.34285
H	10.04849	22.6042	-1.13714
H	10.24713	24.33448	-1.51556
H	10.76411	21.31941	-3.48745
H	14.41537	23.39153	-4.19308
H	12.85262	21.52133	-4.98
H	7.31662	25.28373	1.68207
H	6.55674	26.29475	0.42645
H	7.12596	24.65425	0.02654
H	8.42793	26.61311	-1.78301
H	9.97651	26.00687	2.10035
H	10.87719	27.39855	-1.79088

1-hole SH^- model with 2Asp (B3LYP; tzvp $Cu_4S_2N_7$/sv; PCM=4.0)
0 2

Cu	15.9695	27.55711	1.76019
Cu	12.75043	26.7413	0.96994
Cu	13.6932	25.37288	-1.70069
Cu	15.22099	24.58413	0.37046
S	16.61098	25.36324	2.30761
S	14.67326	26.69678	-0.15078
N	10.84102	26.82347	0.29123
N	9.04306	26.5699	-0.97603
N	12.66792	22.11308	-4.17497
N	12.92804	23.72008	-2.64333
N	14.65004	20.62703	-0.93098
N	14.93876	22.59562	0.04962
N	12.44279	26.54683	3.02602
N	12.20805	27.04802	5.17804
N	14.95042	28.69976	3.43587
N	13.75699	30.195	4.58299
N	16.64422	29.29802	0.89741
N	16.71898	31.012	-0.53
N	13.14751	26.94168	-2.96127
N	12.55001	28.77301	-4.08699
C	10.34778	26.99764	-0.9294
C	9.82238	26.275	1.06069
C	8.69473	26.10925	0.28759
C	7.36496	25.56102	0.61601
C	13.46194	23.12044	-3.72314
C	11.57259	22.04894	-3.34071
C	11.72994	23.04761	-2.39382

C	10.79509	23.40095	-1.29496
C	15.31596	21.82223	-0.96848
C	13.80569	20.64189	0.17123
C	13.98919	21.87066	0.77545
C	13.32991	22.439	1.98394
C	11.82056	27.34211	3.8904
C	13.25285	25.70808	3.77944
C	13.12194	26.00296	5.11919
C	13.79395	25.449	6.31296
C	13.97114	29.58997	3.35908
C	15.40325	28.73584	4.74856
C	14.68122	29.6637	5.47788
C	14.78501	30.121	6.884
C	16.43354	29.68924	-0.36477
C	17.095	30.42796	1.57303
C	17.14007	31.5014	0.6959
C	17.51601	32.92799	0.886
C	13.29111	28.26567	-3.03413
C	12.30579	26.57747	-3.99555
C	11.93459	27.69617	-4.69784
C	11.08801	27.77498	-5.875
H	10.88485	27.39689	-1.78358
H	9.96493	26.02313	2.10674
H	8.43726	26.59815	-1.79054
H	7.12854	24.65413	0.0247
H	6.55238	26.29087	0.42904
H	7.31882	25.28051	1.68169
H	12.97541	21.40807	-4.95937
H	14.407	23.3848	-4.18949
H	10.77962	21.31657	-3.46765
H	10.2158	24.31627	-1.5172
H	10.07076	22.58483	-1.12365
H	11.33028	23.58531	-0.34526
H	14.75134	19.87286	-1.60379
H	16.03426	22.08321	-1.73991
H	13.16049	19.80707	0.42785
H	12.81148	23.38867	1.75231
H	14.06482	22.6485	2.784
H	12.58164	21.7365	2.39047
H	11.10683	28.12317	3.64461
H	13.89941	24.96786	3.32146
H	11.87978	27.5096	6.02037
H	14.42511	24.58913	6.03312
H	14.45336	26.19795	6.79559
H	13.07267	25.10164	7.07841
H	13.4034	29.83417	2.46537
H	16.2171	28.10108	5.09014
H	13.09005	30.93537	4.77659
H	15.06598	31.191	6.95267
H	13.8333	29.9995	7.43858
H	15.55749	29.54385	7.42013

H	16.05601	29.05647	-1.16197
H	17.34278	30.40378	2.63102
H	16.51978	31.56279	-1.44881
H	18.44899	33.18824	0.34679
H	16.72902	33.60716	0.50466
H	17.67571	33.15415	1.95496
H	13.90266	28.91058	-2.41183
H	12.0234	25.54705	-4.18398
H	12.58505	29.75659	-4.34739
H	10.70606	26.77554	-6.14415
H	10.20945	28.43689	-5.73133
H	11.62853	28.16492	-6.76183
H	17.75833	25.30369	1.59519
C	12.16299	18.93701	-6.787
C	13.59632	19.43191	-6.54551
O	14.56594	18.76222	-7.0102
O	13.722	20.53727	-5.86164
C	14.196	33.28501	-3.744
C	14.88901	32.08996	-3.11738
O	14.28993	30.96104	-3.09233
O	16.05912	32.33457	-2.61277
H	11.65481	18.74735	-5.8205
H	11.5754	19.71319	-7.31524
H	13.91077	34.00392	-2.95039
H	14.88664	33.81767	-4.42342
H	13.28879	32.98596	-4.29565
H	12.16479	18.01024	-7.38428

1-hole S^{2-} model with 2Asp (B3LYP; tzvp $Cu_4S_2N_7$/sv; PCM=4.0)
-1 2

Cu	16.73591	27.30878	1.36797
Cu	12.79885	26.76028	0.92329
Cu	13.74679	25.32584	-1.65895
Cu	15.46661	24.56264	0.46862
S	17.00975	25.22421	2.06888
S	14.7622	26.63425	-0.12484
N	10.85395	26.81097	0.28287
N	9.04307	26.5699	-0.97602
N	12.66787	22.11304	-4.17495
N	12.94545	23.69514	-2.61931
N	14.65005	20.62706	-0.93096
N	15.00249	22.56335	0.09664
N	12.55001	26.6517	3.01721
N	12.20805	27.04802	5.17806
N	15.13372	28.93897	3.35403
N	13.75699	30.19498	4.58296
N	17.02782	29.21178	0.75296
N	16.71891	31.01201	-0.52993
N	13.15548	26.93458	-2.96631
N	12.55002	28.77302	-4.08699
C	10.35289	26.99049	-0.93317

C	9.83597	26.26325	1.05488
C	8.70049	26.10449	0.28851
C	7.36496	25.56102	0.61601
C	13.47459	23.1036	-3.70341
C	11.56439	22.05678	-3.35039
C	11.73451	23.03998	-2.38858
C	10.79513	23.40095	-1.29501
C	15.37332	21.79159	-0.92091
C	13.7727	20.66391	0.14551
C	13.99855	21.87157	0.77908
C	13.32991	22.43899	1.98394
C	11.84794	27.36821	3.88701
C	13.39404	25.83821	3.76406
C	13.18573	26.05988	5.11
C	13.79395	25.44901	6.31294
C	14.0463	29.68433	3.32782
C	15.58419	28.96937	4.66474
C	14.74957	29.74722	5.4492
C	14.78503	30.12099	6.88395
C	16.59945	29.65738	-0.43271
C	17.45431	30.34184	1.44772
C	17.26487	31.4709	0.6593
C	17.51605	32.92799	0.88598
C	13.29353	28.25623	-3.03526
C	12.31473	26.57491	-4.00052
C	11.93783	27.69522	-4.69989
C	11.08801	27.77498	-5.875
H	10.89039	27.38541	-1.78942
H	9.9861	26.00784	2.09961
H	8.4356	26.59946	-1.78882
H	7.1202	24.66254	0.015
H	6.55724	26.29947	0.4408
H	7.32092	25.26835	1.67876
H	12.91968	21.493	-5.03352
H	14.42193	23.36564	-4.16631
H	10.75616	21.34504	-3.49758
H	10.23773	24.33002	-1.51593
H	10.05315	22.59734	-1.13776
H	11.32624	23.56972	-0.34031
H	14.73576	19.88139	-1.61414
H	16.12998	22.03264	-1.66184
H	13.08023	19.85685	0.36605
H	12.87332	23.42264	1.76365
H	14.04958	22.5966	2.80945
H	12.53499	21.76364	2.3472
H	11.09785	28.11605	3.64457
H	14.14284	25.20273	3.29561
H	11.82402	27.45278	6.02553
H	14.54263	24.69512	6.01773
H	14.31495	26.19836	6.94188
H	13.04228	24.94446	6.95294

H	13.43236	29.88732	2.45265
H	16.47555	28.42552	4.97273
H	12.99251	30.81908	4.81911
H	14.87287	31.21678	7.0314
H	13.87661	29.79291	7.43003
H	15.653	29.65293	7.37992
H	16.1888	29.03399	-1.22114
H	17.84188	30.27557	2.46049
H	16.45652	31.56612	-1.423
H	18.25635	33.33484	0.16869
H	16.58946	33.52293	0.76424
H	17.9025	33.10585	1.90519
H	13.90351	28.89371	-2.40472
H	12.03578	25.54348	-4.19275
H	12.59168	29.75814	-4.34077
H	10.71237	26.77391	-6.14831
H	10.20415	28.42996	-5.72737
H	11.62276	28.17252	-6.76254
C	12.163	18.93703	-6.787
C	13.42821	19.78692	-6.98195
O	14.26187	19.44778	-7.87501
O	13.55497	20.82272	-6.20122
C	14.19601	33.285	-3.744
C	14.86178	32.06629	-3.14697
O	14.2432	30.94958	-3.15968
O	16.02686	32.28712	-2.62439
H	12.09768	18.58065	-5.73994
H	11.2597	19.54972	-6.97801
H	13.88638	33.97103	-2.93017
H	14.90925	33.84405	-4.37769
H	13.30456	33.01609	-4.33636
H	12.16522	18.07078	-7.46931

2-hole S^{2-} model with 2Asp (B3LYP; tzvp $Cu_4S_2N_7$/sv; PCM=4.0)
0 1

Cu	16.06254	27.52478	1.66593
Cu	12.73057	26.7474	0.97961
Cu	13.6872	25.37735	-1.71368
Cu	15.2112	24.56406	0.31856
S	16.50782	25.38445	2.02254
S	14.67651	26.65154	-0.09578
N	10.84118	26.82428	0.28876
N	9.04298	26.56985	-0.976
N	12.6679	22.11302	-4.17509
N	12.93122	23.7185	-2.64288
N	14.65007	20.62705	-0.93097
N	14.91163	22.60178	0.02662
N	12.45912	26.56582	3.02625
N	12.20801	27.04799	5.17799
N	14.96788	28.71678	3.42953
N	13.757	30.19499	4.58299

N	16.60018	29.3127	0.90089
N	16.719	31.01201	-0.53
N	13.12933	26.95565	-2.93267
N	12.54999	28.77301	-4.08701
C	10.34597	26.99966	-0.9314
C	9.82394	26.27366	1.05993
C	8.69579	26.10779	0.28755
C	7.365	25.56108	0.61601
C	13.4621	23.12071	-3.72621
C	11.57457	22.04826	-3.33896
C	11.7333	23.04569	-2.39083
C	10.79523	23.4011	-1.29482
C	15.29644	21.8283	-0.98689
C	13.81467	20.6351	0.17832
C	13.98124	21.87148	0.77058
C	13.32979	22.43894	1.98395
C	11.82204	27.34808	3.89287
C	13.27558	25.72894	3.7756
C	13.13141	26.01093	5.11661
C	13.794	25.449	6.31298
C	13.98021	29.59658	3.35643
C	15.41752	28.7514	4.74227
C	14.68601	29.66887	5.47603
C	14.78501	30.121	6.884
C	16.41493	29.69683	-0.36666
C	17.05279	30.43747	1.58197
C	17.12525	31.50451	0.69979
C	17.516	32.928	0.886
C	13.28458	28.27824	-3.02613
C	12.28472	26.5833	-3.96604
C	11.92522	27.69503	-4.68684
C	11.08799	27.77501	-5.87502
H	10.88001	27.4021	-1.7855
H	9.96804	26.02197	2.10581
H	8.43548	26.60005	-1.78928
H	7.12602	24.65733	0.02115
H	6.55435	26.29398	0.43308
H	7.31984	25.2764	1.68055
H	12.95254	21.44318	-5.00185
H	14.4028	23.38916	-4.19901
H	10.77924	21.3187	-3.46715
H	10.21627	24.31561	-1.52132
H	10.07063	22.58519	-1.12435
H	11.32671	23.58668	-0.3432
H	14.76244	19.86498	-1.59299
H	16.00684	22.09386	-1.76372
H	13.18861	19.78992	0.44778
H	12.78779	23.37573	1.75345
H	14.07392	22.67514	2.7673
H	12.60411	21.72434	2.40976
H	11.10091	28.12321	3.65

H	13.96385	25.03125	3.31005
H	11.86792	27.49691	6.0225
H	14.45261	24.61215	6.02692
H	14.42434	26.20356	6.82489
H	13.06705	25.06604	7.05608
H	13.40977	29.84014	2.46393
H	16.23557	28.12102	5.08267
H	13.08131	30.92645	4.77994
H	15.05522	31.1935	6.95812
H	13.83468	29.9873	7.43836
H	15.56322	29.5491	7.41756
H	16.04793	29.06424	-1.16873
H	17.28152	30.41295	2.64359
H	16.52537	31.56717	-1.44985
H	18.47554	33.16479	0.38396
H	16.75696	33.61189	0.45991
H	17.63321	33.16786	1.95732
H	13.90084	28.93324	-2.41796
H	11.99586	25.55244	-4.14076
H	12.58805	29.75335	-4.35903
H	11.6451	28.1455	-6.75949
H	10.69138	26.77936	-6.13646
H	10.2209	28.45394	-5.74449
C	12.16302	18.93698	-6.78699
C	13.53922	19.61796	-6.76676
O	14.47817	19.12845	-7.46099
O	13.65052	20.68522	-6.0222
C	14.196	33.28499	-3.744
C	14.88755	32.09734	-3.10133
O	14.28743	30.96893	-3.05928
O	16.05909	32.34631	-2.60192
H	11.85403	18.66249	-5.75897
H	11.39839	19.63579	-7.18012
H	13.92045	34.0193	-2.96128
H	14.88485	33.80162	-4.4374
H	13.28357	32.98086	-4.28401
H	12.18574	18.03174	-7.41585

2-hole SH^- model with 2Asp (B3LYP; tzvp $Cu_4S_2N_7$/sv; PCM=4.0)
1 1

Cu	15.76265	27.66164	1.74807
Cu	12.72241	26.75078	0.99493
Cu	13.66954	25.40454	-1.76279
Cu	15.11444	24.58004	0.29396
S	16.40051	25.46064	2.21048
S	14.62762	26.69306	-0.15971
N	10.85238	26.77985	0.27818
N	9.04299	26.56986	-0.976
N	12.66792	22.11295	-4.17504
N	12.92263	23.74294	-2.66624
N	14.65006	20.62707	-0.93097

N	14.87461	22.62157	-0.00051
N	12.4478	26.5512	3.02729
N	12.208	27.04799	5.17798
N	14.9326	28.69181	3.44185
N	13.757	30.195	4.583
N	16.41388	29.35958	0.92171
N	16.719	31.01201	-0.53
N	13.13618	26.95332	-2.94886
N	12.54998	28.77305	-4.08703
C	10.34925	26.98457	-0.93603
C	9.83584	26.22594	1.04828
C	8.70048	26.08911	0.2816
C	7.36501	25.56107	0.61601
C	13.45241	23.13555	-3.74992
C	11.58562	22.0451	-3.32609
C	11.7353	23.05564	-2.39175
C	10.79525	23.40109	-1.29489
C	15.26272	21.84388	-1.01444
C	13.83416	20.62718	0.19208
C	13.97577	21.87412	0.76729
C	13.3298	22.43893	1.98395
C	11.82532	27.34954	3.89253
C	13.24617	25.70137	3.78294
C	13.11533	25.99828	5.12171
C	13.79401	25.449	6.31299
C	13.96022	29.59599	3.36183
C	15.38854	28.71751	4.75482
C	14.6751	29.65502	5.4804
C	14.785	30.121	6.884
C	16.33101	29.72423	-0.36525
C	16.88271	30.47782	1.61299
C	17.06896	31.51606	0.71355
C	17.516	32.928	0.886
C	13.28781	28.28087	-3.03136
C	12.29147	26.58049	-3.98254
C	11.92854	27.69627	-4.69218
C	11.08799	27.775	-5.87501
H	10.87686	27.40356	-1.78577
H	9.98214	25.95449	2.08864
H	8.42664	26.62681	-1.78172
H	7.09877	24.67909	0.00108
H	6.56801	26.3154	0.46392
H	7.32897	25.24983	1.67316
H	12.97174	21.40339	-4.9854
H	14.38528	23.41024	-4.2346
H	10.80014	21.3019	-3.43452
H	10.20818	24.31088	-1.51851
H	10.07743	22.57872	-1.13057
H	11.32101	23.58354	-0.33908
H	14.77138	19.85998	-1.58661
H	15.95073	22.11618	-1.8088

H	13.2351	19.76973	0.4831
H	12.77809	23.37142	1.7567
H	14.07468	22.66865	2.7692
H	12.60801	21.72252	2.41203
H	11.11443	28.13309	3.64738
H	13.87214	24.94021	3.33323
H	11.87004	27.50238	6.0213
H	14.40297	24.57138	6.03972
H	14.47338	26.19361	6.77354
H	13.07707	25.12993	7.0936
H	13.39854	29.85166	2.46836
H	16.19606	28.07537	5.09582
H	13.0962	30.94107	4.77883
H	15.09134	31.18412	6.94058
H	13.82783	30.02797	7.43265
H	15.54111	29.53097	7.42843
H	15.97744	29.09807	-1.17749
H	17.04616	30.46558	2.68646
H	16.5337	31.57666	-1.46492
H	18.52973	33.09297	0.47032
H	16.83692	33.62445	0.35917
H	17.5427	33.20787	1.95318
H	13.89894	28.93877	-2.4205
H	12.00529	25.55092	-4.16461
H	12.57212	29.75671	-4.35046
H	11.64079	28.15007	-6.75983
H	10.69401	26.77902	-6.13762
H	10.21979	28.45067	-5.73919
H	17.5205	25.51144	1.45287
C	12.163	18.93701	-6.78699
C	13.56828	19.53805	-6.65051
O	14.53666	18.99106	-7.25158
O	13.68061	20.60859	-5.90517
C	14.196	33.28499	-3.744
C	14.86514	32.10786	-3.05845
O	14.24934	30.98979	-2.97489
O	16.04568	32.34964	-2.57072
H	11.75549	18.67927	-5.78946
H	11.4739	19.67774	-7.23821
H	13.97808	34.07327	-2.99714
H	14.87939	33.73288	-4.48877
H	13.25545	32.98578	-4.23547
H	12.18719	18.03244	-7.41614

Electronic Supporting Information References

1. S. Dell'Acqua, S. R. Pauleta, J. J. G. Moura and I. Moura, *Philosophical Transactions of the Royal Society B-Biological Sciences*, 2012, **367**, 1204-1212.
2. E. M. Johnston, S. Dell'Acqua, S. Ramos, S. R. Pauleta, I. Moura and E. I. Solomon, *Journal of the American Chemical Society*, 2014, **136**, 614-617.
3. T. Rasmussen, B. C. Berks, J. N. Butt and A. J. Thomson, *Biochemical Journal*, 2002, **364**, 807-815.
4. M. Prudencio, A. S. Pereira, P. Tavares, S. Besson, I. Cabrito, K. Brown, B. Samyn, B. Devreese, J. Van Beeumen, F. Rusnak, G. Fauque, J. J. G. Moura, M. Tegoni, C. Cambillau and I. Moura, *Biochemistry*, 2000, **39**, 3899-3907.
5. A. Pomowski, W. G. Zumft, P. M. H. Kroneck and O. Einsle, *Nature*, 2011, **477**, 234-U143.
6. K. Brown, K. Djinovic-Carugo, T. Haltia, I. Cabrito, M. Saraste, J. J. G. Moura, I. Moura, M. Tegoni and C. Cambillau, *Journal of Biological Chemistry*, 2000, **275**, 41133-41136.
7. S. Ghosh, S. I. Gorelsky, S. D. George, J. M. Chan, I. Cabrito, D. M. Dooley, J. J. G. Moura, I. Moura and E. I. Solomon, *Journal of the American Chemical Society*, 2007, **129**, 3955-3965.
8. M. J. Frisch, G. W. Trucks, H. B. Schlegel, G. E. Scuseria, M. A. Robb, J. R. Cheeseman, G. Scalmani, V. Barone, B. Mennucci, G. A. Petersson, H. Nakatsuji, M. Caricato, X. Li, H. P. Hratchian, A. F. Izmaylov, J. Bloino, G. Zheng, J. L. Sonnenberg, M. Hada, M. Ehara, K. Toyota, R. Fukuda, J. Hasegawa, M. Ishida, T. Nakajima, Y. Honda, O. Kitao, H. Nakai, T. Vreven, J. A. Montgomery Jr., J. E. Peralta, F. Ogliaro, M. J. Bearpark, J. Heyd, E. N. Brothers, K. N. Kudin, V. N. Staroverov, R. Kobayashi, J. Normand, K. Raghavachari, A. P. Rendell, J. C. Burant, S. S. Iyengar, J. Tomasi, M. Cossi, N. Rega, N. J. Millam, M. Klene, J. E. Knox, J. B. Cross, V. Bakken, C. Adamo, J. Jaramillo, R. Gomperts, R. E. Stratmann, O. Yazyev, A. J. Austin, R. Cammi, C. Pomelli, J. W. Ochterski, R. L. Martin, K. Morokuma, V. G. Zakrzewski, G. A. Voth, P. Salvador, J. J. Dannenberg, S. Dapprich, A. D. Daniels, Ö. Farkas, J. B. Foresman, J. V. Ortiz, J. Cioslowski and D. J. Fox, Gaussian, Inc., Wallingford, CT, USA, 2009.
9. M. D. Hanwell, D. E. Curtis, D. C. Lonie, T. Vandermeersch, E. Zurek and G. R. Hutchison, *Journal of Cheminformatics*, 2012, **4**.
10. W. Humphrey, A. Dalke and K. Schulten, *Journal of Molecular Graphics*, 1996, **14**, 33-38.
11. A. L. Tenderholt, Version 2.3.2 edn.
12. I. Bar-Nahum, A. K. Gupta, S. M. Huber, M. Z. Ertem, C. J. Cramer and W. B. Tolman, *Journal of the American Chemical Society*, 2009, **131**, 2812-+.
13. F. Neese, *Wiley Interdisciplinary Reviews-Computational Molecular Science*, 2012, **2**, 73-78.
